# The Energy Efficiency and the Impact of Air Pollution on Health in China

**DOI:** 10.3390/healthcare8010029

**Published:** 2020-02-03

**Authors:** Xianhui He, Yung-ho Chiu, Tzu-Han Chang, Tai-Yu Lin, Zebin Wang

**Affiliations:** 1 College of International Business, Suzhou Global Institute of Software Technology, Qingshan Road No. 5, Suzhou 215163, China; hexianhui727@126.com (X.H.); szwzb@163.com (Z.W.); 2 Department of Economics, Soochow University 56, Kueiyang St., Sec. 1, Taipei 10048, Taiwan; echiu@scu.edu.tw (Y.-h.C.); angleyc06@gmail.com (T.-H.C.); 3 Department of Business Administration, National Cheng Kung University, No. 1, University Road, Tainan City 701, Taiwan

**Keywords:** data envelopment analysis, dynamic two stage, health efficiency

## Abstract

The rapid growth of China’s economy in recent years has greatly improved its citizens’ living standards, but economic growth consumes many various energy sources as well as produces harmful air pollution. Nitrogen oxides, SO_2_ (sulfur dioxide), and other polluting gases are damaging the environment and people’s health, with a particular spike in incidences of many air pollution-related diseases in recent years. While there have been many documents discussing China’s energy and environmental issues in the past, few of them analyze economic development, air pollution, and residents’ health together. Therefore, this study uses the modified undesirable dynamic two-stage DEA (data envelopment analysis) model to explore the economic, environmental, and health efficiencies of 30 provinces in China. The empirical results show the following: (1) Most provinces have lower efficiency values in the health stage than in the production stage. (2) Among the provinces with annual efficiency values below 1, their energy consumption, CO_2_ (carbon dioxide), and NOx (nitrogen oxide) efficiency values have mostly declined from 2013 to 2016, while their SO_2_ efficiency values have increased (less SO_2_ emissions). (3) The growth rate of SO_2_ efficiency in 2016 for 10 provinces is much higher than in previous years. (4) The health expenditure efficiencies of most provinces are at a lower level and show room for improvement. (5) In most provinces, the mortality rate is higher, but on a decreasing trend. (6) Finally, as representative for a typical respiratory infection, most provinces have a high level of tuberculosis efficiency, indicating that most areas of China are highly effective at respiratory disease governance.

## 1. Introduction

The 2019 China Statistical Yearbook [1] shows that the country’s total energy consumption in 2018 was 4.6 billion tons of standard coal, or 3.41 times that in 1998, with coal and oil consumptions to total energy consumption at 59% and 18.9%, respectively. Fossil fuels such as coal and petroleum produce huge amounts of air pollutants such as sulfur dioxide, nitrogen oxides, soot, and dust. The fine particles contained therein are able to penetrate into human organs such as the respiratory system and the cardiovascular system, thus reducing a person’s immunity and increasing incidences of infectious and chronic diseases. Kim et al. [2] used Meta-AIR (metabolic and asthma incidence research) to analyze the effects of long-term and short-term exposures to air pollution on humans, showing how they are harmful to the human body’s lipid metabolism.

With the largest coal demand in the world, the China government attaches great importance to the prevention and treatment of air pollution. The World Energy Outlook 2017 China Special Report [3] showed that China will continue in its efforts to intensify energy technology innovation, improve the clean and efficient use of fossil energy, accelerate the construction of a clean, low-carbon, safe, and efficient modern energy system, and strive to fulfill its responsibilities and obligations. “The Action Plan for the Comprehensive Management of Air Pollution in the Autumn and Winter of 2019–2020 in the Yangtze River Delta Region” [4] presents the regulation of air pollution in 41 cities at the prefecture level and above in this region for adjusting the industrial structure of heavily polluting industries. Many past research studies have focused on the efficiency of production, energy, environment, treatment and undesirable output analyses, such as [5,6,7,8,9,10,11,12,13,14,15,16,17,18,19,20,21,22,23,24,25,26,27,28,29,30,31,32,33,34,35,36,37,38,39,40,41,42,43]. In addition to energy and environmental efficiency analyses, some scholars have explored the effects of exposure to air pollution on human health, such as [42,43,44,45,46,47].

Past related literature is generally based on static analysis, lacking time considerations and mostly focusing on energy and environmental topics. Moreover, very few studies have jointly investigated economic, environmental, and health efficiencies. When analyzing environment and energy efficiencies, the main consideration is CO_2_, while other just as important pollution indicators are rarely examined. Therefore, this study proposes the modified undesirable dynamic two stage DEA (data envelopment analysis) model to explore economic, environmental, and health efficiencies in China, incorporates other environmental variables like SO_2_ and NO_2_ as outputs, and also employs health variables such as incidences of infectious diseases and tuberculosis as well as dynamic analysis into the model, which make up the contributions of this paper.

**Figure 1:**
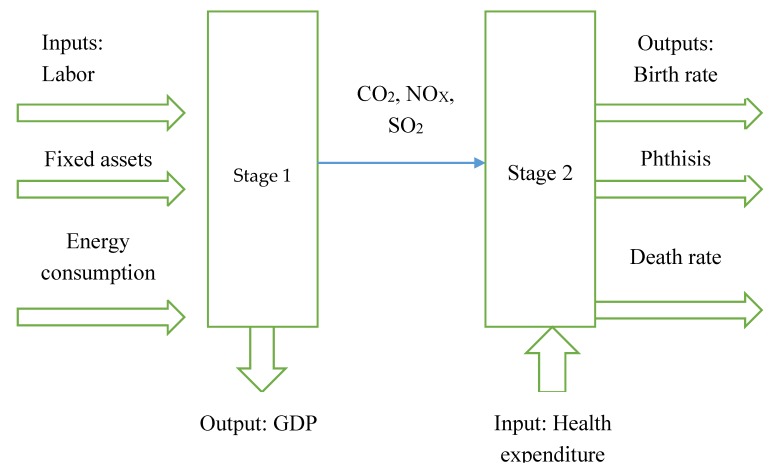
Process of inputs and outputs.

Figure 1 shows the idea behind the input-output relationship and influence mechanism. Our research analyzes the production efficiency and health treatment efficiency of 30 provinces (autonomous regions and municipalities) in China from 2013 to 2016, taking production as the first stage and health treatment as the second stage. In the first stage, energy consumption and labor and capital (capital here indicates fixed assets. In a static model, they are generally used as input in production. In a dynamic model, they are normally used as carry-over, which is capital stock linked to the next period) are inputs for economic growth, which is evaluated by GDP. However, energy consumption generates air pollutants, such as CO_2_, SO_2_, and NO_x_, that increase death rates and phthisis. The government thus needs to use health expenditure to reduce the negative effects of air pollutants. In the second stage, health expenditure is the input, the desirable output is the birth rate, and the undesirable outputs are the death rate and phthisis.

The remainder of this paper is organized as follows. Section 2 is the literature review. Section 3 shows the research method. Section 4 presents the empirical results and discussion. Section 5 is the conclusion.

## 2. Literature Review

Many research studies have focused on energy, environmental pollution, and human health issues. The literature discussed in this section can be divided into three directions as noted below.

(a) The literature that uses the two-stage data envelopment analysis (DEA) method to analyze the efficiencies of production, environment, undesirable output, and treatment

Wu et al. [5] used the two-stage network DEA model to explore China’s emission reduction efficiency from 2006 to 2010, presenting results that energy-saving and emission reduction effects are the best in the eastern region. Chen et al. [6] employed the modified Undesirable Dynamic Network model to analyze the efficiencies of China’s energy emissions, environment, health, and media communication, showing results that the production efficiency stage of its cities is better than the health treatment stage. Li et al. [7] utilized the two-stage DEA model to explore ecological and wastewater treatment efficiencies in China’s provinces, presenting that most provinces have low ecological efficiency and need to improve their efficiency values. Wu et al. [8] used the two-stage network DEA model to analyze the industrial production and environmental efficiencies of 30 provinces of China in 2010. The research results noted that the environmental efficiency of most provinces needs improvement. Iftikhar et al. [9] took the network DEA model to analyze the energy and emission reduction efficiencies of China and the United States, presenting results that overall efficiency is low, and that 85% of energy consumption and 89% of CO_2_ emissions are caused by low economic and allocation efficiency. Zhou et al. [10] used a mixed network structure two-stage SBM DEA model to explore China’s environmental water efficiency from 2006 to 2015. The results showed that water use efficiency is higher than wastewater treatment efficiency, and thus the latter need to be improved. A province with good economic development and social progress also has higher efficiency.

Li et al. [11] employed two-stage data envelopment analysis to analyze China’s environmental treatment efficiency from 2006 to 2015. Findings show that industrial waste treatment efficiency remained stable for five years, and that the efficiency of the pollution disposal stage was higher than that of the resource reuse stage. Li et al. [12] used the network slack-based measure (DNSBM) model to explore the energy and air pollution efficiencies of 30 provinces in China from 2013 to 2016. The results denoted that the provinces exhibit low efficiency and great differences, and that the level of air pollution control in the second stage is low. Stefaniec [13] utilized the dynamic two-stage SBM DEA model to analyze Iranian refinery production and environmental efficiency from 2011 to 2015. Results showed that most Iranian refineries are inefficient. Yongqi et al. [14] utilized the two-stage meta-frontier dynamic network data envelopment analysis (TMDN-DEA) model to explore the production and environmental efficiencies of 28 EU countries from 2010 to 2014. Their findings showed that the average annual overall efficiency of EU countries is higher than that of non-EU countries, and EU countries have higher energy efficiency. EU countries and non-EU countries also have higher PM_2.5_ efficiency than CO_2_ efficiency.

Zhang et al. [15] used the two-stage DEA model to explore the environmental efficiency of 30 provinces in China from 2011 to 2015, noting that most provinces have low emissions efficiency and need to improve this. Li et al. [11] took two-stage data envelopment analysis to look into China’s environmental treatment efficiency from 2006 to 2015, stating that industrial waste treatment efficiency remained stable for five years. The efficiency of the pollution disposal stage is higher than that of the resource reuse stage. Zhai et al. [16] used two-stage frontier-shift data envelopment analysis on China’s energy supply chain and emission reduction efficiency. The results showed that when the city’s average carbon credits traded to competitors were 392.21 tons, it would increase energy efficiency by 6.61%. Xuancheng ranks first in emission reduction efficiency, and Fuyang needs to improve its efficiency performance. Shao et al. [17] employed a two-stage directional distance function to explore the environmental eco-efficiency of various industries in China from 2007 to 2015. The results showed that exhaust gas treatment efficiency performs best, followed by wastewater treatment and production efficiencies. China’s industrial, electric power, and natural gas production and supply sectors have the highest ecological efficiency, while the mining industry has the lowest ecological efficiency.

(b) The literature that analyzes energy and environmental efficiency

Borozan and Djula [18] used the two-stage DEA model to explore EU regions’ technology and energy efficiencies from 2005 to 2013. The results showed that the regional differences in technology and energy efficiencies are quite large, and that total factor energy efficiency is low in most EU regions. Zhu et al. [19] utilized network data envelopment analysis on the production and ecological efficiencies of Chinese provinces, presenting findings that the ecological efficiency of each province is low. Jebali et al. [20] used the two-stage double bootstrap DEA approach to explore the energy efficiency of Mediterranean countries from 2009 to 2012, stating that energy efficiency in these countries is high. Per capita gross national income, population density, and renewable energy use could affect energy efficiency performance. Mavi et al. [21] used two-stage network DEA to analyze OECD production and ecological efficiency, offering evidence that Switzerland has the highest ecological efficiency and Estonia had the highest ecological innovation.

For the first literature strand on energy emissions and environmental efficiencies, Choi et al. [22] employed the SBM-DEA method to explore the energy efficiency of various provinces in China and found that carbon dioxide efficiency is not good. Li and Hu [23] used the SBM DEA model to analyze the ecological total-factor energy efficiency (ETFEE) of 30 regions in China from 2005 to 2009, showing results that regional energy efficiency is uneven, and that the eastern region has the best emissions efficiency. Song et al. [24] found that Brazil, Russia, India, and China (BRIC) have lower energy emission efficiency. Yang and Wang [25] used the DEA model to explore the energy and emissions efficiencies of various provinces in China from 2000 to 2007. Their results showed that the energy and emissions efficiencies of China’s provinces are very different. Zhang and Choi [26] employed SBM-DEA to analyze the energy and environmental efficiencies in China, with most provinces exhibiting inefficiency. Wang et al. [27] used the directional distance function model to explore the CO_2_ emission efficiencies of various provinces in China, showing that the CO_2_ emissions of provinces in the southeastern coastal areas are relatively high. Bi et al. [28] employed the DEA model to explore the energy and environmental efficiencies of thermal power generation in China, with findings that energy efficiency and environmental efficiency are relatively low.

Lin and Du [29] utilized the non-radial directional distance function to assess regional energy and carbon dioxide emissions efficiencies in China from 1997 to 2009, noting that energy use and carbon dioxide emissions performances in most parts of China are poor. Meng et al. [30] explored the energy efficiency and carbon emission efficiency of China’s provinces from 2006 to 2015, finding that these two efficiencies in the eastern region are excellent, while in the central region they are the worst. Yao et al. [31] used the meta-frontier non-radial Malmquist CO_2_ emission performance index (MNMCPI) indicator to analyze the CO_2_ emission efficiency of China’s industrial sector from 1998 to 2011. The results showed that the country’s annual growth rate of CO_2_ emissions from the provincial industrial sector is 5.53%. Feng et al. [32] utilized the three-hierarchy meta-frontier data envelopment model to analyze the emission efficiency of 30 provinces in China, noting that China’s carbon dioxide emissions efficiency is relatively low. Jebali et al. [20] studied the effects of energy efficiency emissions in Mediterranean countries from 2009 to 2012, presenting that these countries have high energy efficiency standards.

Du et al. [33] explored CO_2_ emission efficiencies in China’s provinces from 2006 to 2012. Their findings are that economic activity (EAT) is the main driver of increased emissions, while potential energy intensity (PEI) changes, energy structure changes (EMX), and energy efficiency changes (EC) reduce CO_2_ emissions in most provinces of China. Wang et al. [34] used the meta-frontier DEA model to explore the carbon emission efficiency of different countries, presenting results that the overall carbon emissions of Asia are lower than those of Europe and U.S. Qin et al. [35] employed the directional distance function model to explore the energy and environmental efficiencies of China’s coastal areas from 2000 to 2012. The results presented that the level of economic development has a positive relationship with energy efficiency. Wang et al. [36] showed a negative correlation between resource richness and carbon emission efficiency in China from 2003 to 2016.

Li et al. [37] used the dynamic DEA model to analyze the energy and emissions efficiencies of 31 cities in China from 2013 to 2016, stating that Beijing, Guangzhou, Shanghai, Lhasa, and Nanning have the best emissions efficiency. Li et al. [38] employed the meta frontier dynamic Data Envelopment Analysis model to explore energy and emissions efficiencies in OECD countries and non-OECD countries from 2010 to 2017. The results exhibited that the efficiencies of the United Arab Emirates and Singapore are increasing year by year. Ren et al. [39] used the meta-frontier dynamic SBM model to explore the energy and emissions efficiencies of the Yangtze River Economic Belt (YREB) in China from 2014 to 2016. Their results showed that YREB’s energy and emissions efficiencies are higher than those of non-YREB. Using the non-radial directional distance function model, Teng et al. [40] found that China’s energy efficiency has great room for improvement. Zhou et al. [41] used the Super-SBM DEA model to analyze the emissions efficiency of China’s construction industry from 2003 to 2016, noting that this industry has low carbon emissions.

Decision making is important for efficiency analysis, Kou et al. [42] used multiple criteria decision-making (MCDM) methods to classify small samples, and confirmed that the MCDM method can be effectively used for evaluation. Li et al. [43] used the group decision-making (GDM) method for integrating heterogeneous information and applied it to a numerical example of supplier selection. Zhang et al. [44] analyzed cost issue by GDM. Kou et al. [45] reviewed and classified 37 main literatures of pairwise comparison matrix (PCM) from 2010 to 2015, which helped to use PCM in measurements. Kou et al. [46] proposed an approach to resolve disagreements among MCDM methods based on Spearman’s rank correlation coefficient, and the results showed that the differences between MCDM rankings were greatly reduced. Kou et al. [47] used multiple criteria decision making (MCDM) to analyze financial credit and bankruptcy risk, and the results showed that MCDM was effective in evaluating clustering algorithms.

(c) The literature on the negative effect of air pollutants on human health

Schiavon et al. [48] used the AUSTAL2000 dispersion model to show that high concentrations of emissions that have occurred in Italy affect the human body. Fischer et al. [49] showed that long-term exposure to PM_10_ and NO_2_ is associated with mortality in the Netherlands. Lelieveld et al. [50] confirmed that the annual premature mortality rate of 3.3% globally is due to outdoor air pollution, mainly in Asian countries. Li et al. [51] explored the effects of exposure to air pollution on public health. Their results showed that PM_10_ and SO_2_ cause serious economic losses and increased human health-related problems. Wu et al. [52] studied the relationship between antioxidant activity and ambient air pollution, presenting that exposure to particulate air pollution can affect the body’s circulating antioxidant enzymes. Yang et al. [53] noted that long-term exposure to ambient air pollution has an impact on hypertension. Vlaanderen et al. [54] explored how short-term exposure to air pollution affects human blood metabolism. Shen et al. [55] introduced the total air quality index (AAQI) and health risk air quality index (HAQI) to assess human health risks. According to the HAQI results, the current AQI system may significantly underestimate the health risks of air pollution, and the public may need stricter health protection measures to reduce their risk. Zhao et al. [56] explored the problems caused by air pollution in Beijing, China in 2015, which has a negative impact on the health of cyclists.

Dauchet et al. [57] explored two urban areas in northern France, presenting that an increase in O3 is associated with a rise in the blood eosinophil count. Kasdagli et al. [58] used a systematic review and meta-analysis to analyze the relationship between air pollution exposure and Parkinson’s disease (PD), noting that there is insufficient evidence of air pollution and PD caused by traffic. Ljungmau et al. [59] used linear regression to analyze the relationship between long-term and short-term air pollution exposure and arterial stiffness, and their results showed that long-term exposure to PM_2.5_ is not associated with arterial stiffness. Ngo et al. [60] employed hospitalization and emergency department data to analyze the incidences of patients with respiratory diseases and heart disease in California between 2005 and 2012. Heavy exposure to sandstorms is associated with an increase in human acute respiratory illness. Torres et al. [61] studied the adverse effects of exposure to air pollution in Alentejo and the Lisbon metropolitan area on human health. Their results showed that cardiovascular and respiratory disease-related mortalities occur mainly in the Alentejo and Algarve regions. Khaniabadi et al. [62] looked into human health problems caused by exposure to air pollution in developing countries. Exposure to air pollution caused a cardiovascular disease (CM) mortality of 4.96%, respiratory diseases (HA-RD) of 4.97%, cardiovascular disease (HA-CVD) of 5.55%, lung disease (HA-COPD) of 2.50%, and acute myocardial infarction (AMI) of 4.73%. Bayat et al. [63] used the Environmental Benefits Mapping and Analysis Program (BenMAP-CE) to explore the human health problems caused by exposure to air pollution in Tehran, Iran. The results showed that a total of 7146 adults with PM_2.5_ exposure died of ischemic heart disease, stroke, lower respiratory tract infection, chronic obstructive pulmonary disease, and lung cancer. Huang et al. [64] examined the relationship between PM_2.5_ and the social economy in Beijing, China, with household income and education negatively correlated with environmental air quality in 2014. Lua et al. [65] used a 1 km high-resolution annual satellite search for PM_2.5_ data to analyze the concentration of PM_2.5_ in China from 2001 to 2017 and its adverse health effects.

## 3. Materials and Methods

### 3.1. DEA Basis

DEA is a linear programming model that evaluates a decision making unit (DMU) based on Pareto’s optimal solution, instead of finding the efficiency frontier with a preset function as an analysis of the relative efficiency relationship between DMUs. Farrell [66] used the frontier production function to measure the level of production efficiency of DMUs, connecting the most efficient production points to the theoretical production frontier. Here, the gap between any real production point and the theoretical production frontier indicates the degree of inefficient production. However, the problem of Farrell’s theoretical model is that it can only be applied to a single input and a single output; it cannot meet the actual benefits of presenting multiple inputs and multiple outputs. Charnes et al. [67] proposed the CCR model in 1978, extending the Farrell model into a generalized mathematical linear programming model that can be used to measure the performance evaluation of multiple inputs and multiple outputs of constant returns to scale, naming it the data envelopment analysis method (DEA). Banker et al. [68] proposed the BCC model in 1984, revising the assumption of constant returns to scale to variable returns to scale (VRS). Since the CCR model and the BCC model measure radial efficiency, these two models assume that the input or output can be adjusted (increased or decreased) in equal proportions, but this assumption is not applicable in some cases. Tone [69] thus proposed the Slacks-Based Measure (SBM) model in 2001, which uses the difference variable as the basis for measurement, taking into account the difference between the input and output items (slack) and using the non-radial estimation method and a single value (scalar) to present SBM efficiency.

The traditional DEA model uses input and output projections to perform an efficiency conversion between two variables, and the conversion process is identified as a “black box”. Fare et al. [70] proposed the Network DEA model, noting that the production process is composed of many sub-production technologies, and regarded the sub-production technology as a sub-DMU that can be solved by the traditional CCR and BCC models. Zhu [71] described the value chain process as a “black box” and believed that it must contain some sub-processes that constitute the value chain system. To estimate the efficiency of a system, it is necessary to evaluate the efficiency of each of these sub-processes. Chen and Zhu [72], Kao and Hwang [73], and Kao [74] divided the whole business process into sub-processes and connected the stages through some intermediate outputs. They calculated the efficiency of each stage under different conditions and analyzed which sub-process caused efficiency loss to the system. Tone and Tsutsui [75] proposed the weighted slack-based measures network data envelopment analysis model. The linkage between the departments of the decision-making unit is used as the basis for the analysis of the Network DEA model, and each department is regarded as a sub-DMU, and thus one can use the SBM model to find an optimal solution. In the network DEA model, more and more studies in the literature are devoted to the research of the multi-stage production process and its efficiency evaluation. Castelli et al. [76] reviewed multi-stage models. The two-stage DEA also takes a dynamic approach in which DMUs are evaluated at different time periods and carry-overs are introduced to connect the various stages.

### 3.2. Measures Network DEA

Tone and Tsutsui [75] proposed the slack-based measures network DEA model, which is used to measure the overall efficiency of decision-making units and the efficiency of various departments. The SBM model is a non-radial measurement method that is suitable for the inputs and outputs that cannot be adjusted in equal proportions. The following part describes the non-oriented network DEA model, indicating the objective function, the department efficiency, and the solving process of a decision-making unit.

Non-oriented model:

If the input slack and output slack are considered at the same time, then non-oriented efficiency can be evaluated by Formula (1):

(1) Min ρo*=∑k=1Kwk[1−1mk(∑i=1mksiok−Xiok)]∑k=1Kwk[1+1rk(∑r=1rksrok+Yrok)]∑k=1Kwk=1,∀kwk≥0,∀k

According to (1), the definition of non-oriented department efficiency can be expressed as:

(2) ρk=1−1mk(∑i=1mksiok−*Xiok)1+1rk(∑r=1rksrok+*Yrok),k=1,2,……,K

Here, $s_{io}^{k-*}$ and $s_{ro}^{k+*}$ are the optimal input slack and the optimal output slack, respectively. If $\rho_{k}^{*}=1$, then $DMU_{o}$ ’s k department has non-oriented efficiency; if $\rho_{o}^{*}=1$, then $DMU_{o}$ has non-oriented overall efficiency. Whether it is input-oriented, output-oriented, or non-oriented, the assessed departmental efficiency and the overall efficiency of decision-making units have unit invariance.

Traditional DEA cannot analyze the efficiency of individual departments, and so we need to use Network DEA. At the same time, a company’s operations might span several periods. Thus, a dynamic DEA model must be used. If one considers departments and time, then a combination of Network DEA and dynamic DEA is needed.

Many scholars research on Dynamic DEA such as Kloop [77], Malmquist [78], Fare et al. [79] Malmquist index. These research studies did not analyze the “the effect of carry-over activities” in two periods. Fare and Grosskopf [80] were the first to put inter-connecting activities into the dynamic model. Nemotoa and Goto [81] added some important insights on dynamic DEA, while Nemoto and Goto [82] proposed a method using dynamic DEA to instantly adjust to a quasi-fixed input at the optimal level. Sueyoshi and Sekitani [83] incorporated the concept of returns to scale into a dynamic DEA model. Amirteimoori [84] defined the DEA model to assess dynamic income efficiency, which was modified and extended by Färe and Grosskopf [80]. Tone and Tsutsui [85] extended the model to a dynamic analysis of slacks-based measures. Tone and Tsutsui [86] proposed the slack-based measures dynamic network DEA model. The linkage between various departments of the decision-making unit is used as the basis for the analysis of the network DEA model, and each department is regarded as a sub-DMU, and carry-over activities are used as the linkage. Carry-over activities can be divided into 4 types: (1) desirable, (2) undesirable, (3) discretionary, and (4) non-discretionary. We introduce the dynamic two-stage DEA model as follows.

Non-oriented model:

(a) Overall efficiency:
  (3) $$\theta_{0}^{*}=\min\frac{\sum_{t=1}^{T}W^{t}\left[\sum_{k=1}^{K}W^{k}\left[1-\frac{1}{m_{k}+linkin_{k}+nbad_{k}}(\sum_{i=1}^{m_{k}}\frac{S_{iok}^{t-}}{x_{iok}^{t}}+\sum_{{\left(kh\right)}_{l}=1}^{linkin_{k}}\frac{s_{o{\left(kh\right)}_{l}in}^{t}}{z_{o{\left(kh\right)}_{l}in}^{t}}+\sum_{k_{l}=1}^{nbad_{k}}\frac{s_{ok_{l}bad}^{\left(t,(t+1)\right)}}{z_{ok_{l}bad}^{\left(t,(t+1)\right)}})\right]\right]}{\sum_{t=1}^{T}W^{t}\left[\sum_{k=1}^{K}W^{k}\left[1+\frac{1}{r_{k}+linkout_{k}+ngood_{k}}(\sum_{r=1}^{r_{k}}\frac{s_{rok}^{t+}}{y_{rok}^{t}}+\sum_{{\left(kh\right)}_{l}=1}^{linkout_{k}}\frac{s_{o{\left(kh\right)}_{l}out}^{t}}{z_{o{\left(kh\right)}_{l}out}^{t}}+\sum_{k_{l}=1}^{ngood_{k}}\frac{s_{ok\mathrm{lg}ood}^{\left(t,(t+1)\right)}}{z_{ok\mathrm{lg}ood}^{\left(t,(t+1)\right)}})\right]\right]}$$
  Constraint (4)
  (4) $$x_{ok}^{t}=X_{k}^{t}\lambda_{k}^{t}+s_{ko}^{t-}\left(\forall k,\forall t\right)\;y_{ok}^{t}=Y_{k}^{t}\lambda_{k}^{t}-s_{ko}^{t+}\left(\forall k,\forall t\right)\;e\lambda_{k}^{t}=1\left(\forall k,\forall t\right)\;\lambda_{k}^{t}\geq 0,s_{ko}^{t-1}\geq 0,s_{ko}^{t+}\geq 0,\left(\forall k,\forall t\right)\;Z_{(kh)free}^{t}\lambda_{h}^{t}=Z_{(kh)free}^{t}\lambda_{k}^{t}\left(\forall (k,h)free,\forall t\right)\;Z_{(kh)free}^{t}=(Z_{1(kh)free}^{t},\ldots ,Z_{n(kh)free}^{t})\in R^{L_{(h)free}\times n}\;Z_{o(kh)fix}^{t}=Z_{(kh)fix}^{t}\lambda_{h}^{t}\left(\forall (k,h)fix,\forall t\right)\;Z_{o(kh)fix}^{t}=Z_{(kh)fix}^{t}\lambda_{k}^{t}\left(\forall (k,h)fix,\forall t\right)\;Z_{o(kh)in}^{t}=Z_{(kh)in}^{t}\lambda_{k}^{t}+S_{o(kh)in}^{t}\left((kh)in=1,\ldots ,linkin_{k}\right)\;Z_{o(kh)out}^{t}=Z_{(kh)out}^{t}\lambda_{k}^{t}-S_{o(kh)out}^{t}\left((kh)out=1,\ldots ,linkout_{k}\right)\;\sum_{j=1}^{n}z_{jk_{1}\alpha }^{\left(t,(t+1)\right)}\lambda_{jk}^{t}=\sum_{j=1}^{n}z_{jk_{1}\alpha }^{\left(t,(t+1)\right)}\lambda_{jk}^{t+1}\left(\forall k;\forall k_{l};t=1,\ldots T-1\right)\;Z_{ok_{l}good}^{\left(t,(t+1)\right)}={\sum_{j=1}^{n}z}_{jk_{l}good}^{\left(t,(t+1)\right)}\lambda_{jk}^{t}-s_{ok_{l}good}^{\left(t,(t+1)\right)}k_{l}=1,\ldots ,ngood_{k};\forall k;\forall t)\;Z_{ok_{l}bad}^{\left(t,(t+1)\right)}={\sum_{j=1}^{n}z}_{jk_{l}bad}^{\left(t,(t+1)\right)}\lambda_{jk}^{t}-s_{ok_{l}bad}^{\left(t,(t+1)\right)}k_{l}=1,\ldots ,nbad_{k};\forall k;\forall t)\;Z_{ok_{l}free}^{\left(t,(t+1)\right)}={\sum_{j=1}^{n}z}_{jk_{l}free}^{\left(t,(t+1)\right)}\lambda_{jk}^{t}-s_{ok_{l}free}^{\left(t,(t+1)\right)}k_{l}=1,\ldots ,nfree_{k};\forall k;\forall t)\;Z_{ok_{l}fix}^{\left(t,(t+1)\right)}={\sum_{j=1}^{n}z}_{jk_{l}fix}^{\left(t,(t+1)\right)}\lambda_{jk}^{t}-s_{ok_{l}fix}^{\left(t,(t+1)\right)}k_{l}=1,\ldots ,nfix_{k};\forall k;\forall t)\;s_{ok_{l}good}^{\left(t,(t+1)\right)}\geq 0,s_{ok_{l}bad}^{\left(t,(t+1)\right)}\geq 0,s_{ok_{l}free}^{\left(t,(t+1)\right)}:free(\forall k_{l};\forall t)$$
(b) Period and division efficiencies:
  (b1) Period efficiency:
    (5) $$\tau_{0}^{t*}=\frac{\sum_{k=1}^{K}W^{k}\left[1-\frac{1}{m_{k}+linkin_{k}+nbad_{k}}(\sum_{i=1}^{m_{k}}\frac{S_{iok}^{t-}}{x_{iok}^{t}}+\sum_{{\left(kh\right)}_{l}=1}^{linkin_{k}}\frac{s_{o{\left(kh\right)}_{l}in}^{t}}{z_{o{\left(kh\right)}_{l}in}^{t}}+\sum_{k_{l}=1}^{nbad_{k}}\frac{s_{ok_{l}bad}^{\left(t,(t+1)\right)}}{z_{ok_{l}bad}^{\left(t,(t+1)\right)}})\right]}{\sum_{k=1}^{K}W^{k}\left[1+\frac{1}{r_{k}+linkout_{k}+ngood_{k}}(\sum_{r=1}^{r_{k}}\frac{s_{rok}^{t+}}{y_{rok}^{t}}+\sum_{{\left(kh\right)}_{l}=1}^{linkout_{k}}\frac{s_{o{\left(kh\right)}_{l}out}^{t}}{z_{o{\left(kh\right)}_{l}out}^{t}}+\sum_{k_{l}=1}^{ngood_{k}}\frac{s_{ok_{l}good}^{\left(t,(t+1)\right)}}{z_{ok_{l}good}^{\left(t,(t+1)\right)}})\right]}$$
  (b2) Division efficiency:
    (6) $$\delta_{0k}^{*}=\frac{\sum_{t=1}^{T}W^{t}\left[1-\frac{1}{m_{k}+linkin_{k}+nbad_{k}}(\sum_{i=1}^{m_{k}}\frac{S_{iok}^{t-}}{x_{iok}^{t}}+\sum_{{\left(kh\right)}_{l}=1}^{linkin_{k}}\frac{s_{o{\left(kh\right)}_{l}in}^{t}}{z_{o{\left(kh\right)}_{l}in}^{t}}+\sum_{k_{l}=1}^{nbad_{k}}\frac{s_{ok_{l}bad}^{\left(t,(t+1)\right)}}{z_{ok_{l}bad}^{\left(t,(t+1)\right)}})\right]}{\sum_{t=1}^{T}W^{t}\left[1+\frac{1}{r_{k}+linkout_{k}+ngood_{k}}(\sum_{r=1}^{r_{k}}\frac{s_{rok}^{t+}}{y_{rok}^{t}}+\sum_{{\left(kh\right)}_{l}=1}^{linkout_{k}}\frac{s_{o{\left(kh\right)}_{l}out}^{t}}{z_{o{\left(kh\right)}_{l}out}^{t}}+\sum_{k_{l}=1}^{ngood_{k}}\frac{s_{ok_{l}good}^{\left(t,(t+1)\right)}}{z_{ok_{l}good}^{\left(t,(t+1)\right)}})\right]}(\forall k)$$
  (b3) Division period efficiency:
    (7) $$p_{0k}^{t*}=\frac{1-\frac{1}{m_{k}+linkin_{k}+nbad_{k}}(\sum_{i=1}^{m_{k}}\frac{S_{iok}^{t-}}{x_{iok}^{t}}+\sum_{{\left(kh\right)}_{l}=1}^{linkin_{k}}\frac{s_{o{\left(kh\right)}_{l}in}^{t}}{z_{o{\left(kh\right)}_{l}in}^{t}}+\sum_{k_{l}=1}^{nbad_{k}}\frac{s_{ok_{l}bad}^{\left(t,(t+1)\right)}}{z_{ok_{l}bad}^{\left(t,(t+1)\right)}})}{1+\frac{1}{r_{k}+linkout_{k}+ngood_{k}}(\sum_{r=1}^{r_{k}}\frac{s_{rok}^{t+}}{y_{rok}^{t}}+\sum_{{\left(kh\right)}_{l}=1}^{linkout_{k}}\frac{s_{o{\left(kh\right)}_{l}out}^{t}}{z_{o{\left(kh\right)}_{l}out}^{t}}+\sum_{k_{l}=1}^{ngood_{k}}\frac{s_{ok_{l}good}^{\left(t,(t+1)\right)}}{z_{ok_{l}good}^{\left(t,(t+1)\right)}})}(\forall k;\forall t)\;Z_{ol_{k}}^{\left(0,1\right)}=\sum_{j=1}^{n}Z_{jlk}^{\left(0,1\right)}\lambda_{jk}^{l}\left(\forall l_{k}\right)$$

This model treats undesirable output as a two-stage link or carry-over link. In this paper, referring to Tone and Tsutsui [75], the output of the SBM model is divided into desirable output and desirable output. Based on the Tone and Tsutsui [86] dynamic network DEA model, a Modified Undesirable Dynamic Network Model is proposed.

### 3.3. Modified Undesirable Dynamic Network Model

Our model has two stages. Production is the first stage, and health treatment is the second stage. At the production stage, labor and energy consumed are the inputs, and GDP is the output indicator. CO_2_, SO_2_, and NO_x_ are the variables linking production with health treatment. The health treatment stage uses health expenditures as input, and the outputs are birth rate, death rate, and phthisis. The carry-over variable is fixed assets.

Suppose there are n DMUs (*j* = 1,…,n), with each having k divisions (*k* = 1,…,K), and there are T time periods (*t* = 1,…,T). Each DMU has an input and output at time period *t* and a carryover (link) to the next *t* + 1 time period.

We set *m_k_* and *u_k_* to represent the inputs and outputs in each division K, with (*k*,*h*)*_i_* representing divisions *k* to *h* and *Lhk* being the *k* and *h* division sets. The inputs, outputs, links, and carry-over definitions are outlined in the following paragraphs. The following is the non-oriented model.

(a) Objective function:
  Overall efficiency:
  $$\theta_{0}^{*}=\min\frac{\sum_{t=1}^{T}W^{t}\left[\sum_{k=1}^{K}W^{k}\left[1-\frac{1}{m_{k}+linkin_{k}+ninput_{k}}(\sum_{i=1}^{m_{k}}\frac{S_{iok}^{t-}}{x_{iok}^{t}}+\sum_{{\left(kh\right)}_{l}=1}^{linkin_{k}}\frac{s_{o{\left(kh\right)}_{l}in}^{t}}{z_{o{\left(kh\right)}_{l}in}^{t}}+\sum_{k_{l}}^{ninput_{k}}\frac{s_{ok_{l}input}^{\left(t,t+1\right)}}{z_{ok_{l}input}^{\left(t,t+1\right)}})\right]\right]}{\sum_{t=1}^{T}W^{t}\left[\sum_{k=1}^{K}W^{k}\left[1+\frac{1}{r_{1k}+r_{2k}}(\sum_{r=1}^{r_{1k}}\frac{s_{rokgood}^{t+}}{y_{rokgood}^{t}}+\;\sum_{r=1}^{r_{2k}}\frac{s_{rokbad}^{t-}}{y_{rokbad}^{t}})\right]\right]}$$
  Subject to:
  (8) $$\begin{array}{l} x_{ok}^{t}=X_{k}^{t}\lambda_{k}^{t}+s_{ko}^{t-}\left(\forall k,\forall t\right)\\ y_{okgood}^{t}=Y_{kgood}^{t}\lambda_{k}^{t}-s_{kogood}^{t+}\left(\forall k,\forall t\right)\\ y_{okbad}^{t}=Y_{kbad}^{t}\lambda_{k}^{t}+s_{kobad}^{t-}\left(\forall k,\forall t\right)\\ e\lambda_{k}^{t}=1\left(\forall k,\forall t\right)\\ \lambda_{k}^{t}\geq 0,s_{ko}^{t-}\geq 0,s_{kogood}^{t+}\geq 0,s_{kobad}^{t-}\geq 0,\left(\forall k,\forall t\right)\\ Z_{o(kh)in}^{t}=Z_{(kh)in}^{t}\lambda_{k}^{t}+S_{o(kh)in}^{t}\left((kh)in=1,\ldots ,linkin_{k}\right)\\ \sum_{j=1}^{n}z_{jk_{1}\alpha }^{\left(t,(t+1)\right)}\lambda_{jk}^{t}=\sum_{j=1}^{n}z_{jk_{1}\alpha }^{\left(t,(t+1)\right)}\lambda_{jk}^{t+1}\left(\forall k;\forall k_{l};t=1,\ldots ,T-1\right)\\ Z_{ok_{l}input}^{\left(t,(t+1)\right)}={\sum_{j=1}^{n}z}_{jk_{l}input}^{\left(t,(t+1)\right)}\lambda_{jk}^{t}+s_{ok_{l}input}^{\left(t,(t+1)\right)}k_{l}=1,\ldots ,ngood_{k};\forall k;\forall t)\\ s_{ok_{l}good}^{\left(t,(t+1)\right)}\geq 0,\left(\forall k_{l};\forall t\right) \end{array}$$
  where $W^{t}$ (∀ *t*) is the weight to period *t* and $W^{k}$ (∀ . *k*) is the weight to division *k*. These weights satisfy the condition: $\overset{K}{\underset{k=1}{\sum }}W^{k}=1$ $\overset{T}{\underset{t=1}{\sum }}W^{t}=1.\;$ They are supplied exogenously. They are weighted by the divisional weight $W^{k}$ and further by the period weight $W^{t}$, and result in the overall-efficiency $\theta_{0}^{*}$. This objective function is a generalization of the slacks-based measure (SBM) developed in Tone and Tsutsui [80]. In the Equation (8), the overall efficiency is uniquely determined. However, the period efficiency may not be uniquely determined, so we use priority principle scheme to solve the non-uniqueness problem (Tone and Tsutsui [80]).
(b) Period and division efficiencies:
  Period and division efficiencies are as follows.
  (b1) Period efficiency:
    (9) $$\partial_{0}^{*}=\min\frac{\sum_{k=1}^{K}W^{k}\left[1-\frac{1}{m_{k}+linkin_{k}}(\sum_{i=1}^{m_{k}}\frac{S_{iok}^{t-}}{x_{iok}^{t}}+\sum_{{\left(kh\right)}_{l}=1}^{linkin_{k}}\frac{s_{o{\left(kh\right)}_{l}in}^{t}}{z_{o{\left(kh\right)}_{l}in}^{t}})\right]}{\sum_{k=1}^{K}W^{k}\left[1+\frac{1}{r_{1k}+r_{2k}+ngood_{k}}(\sum_{r=1}^{r_{1k}}\frac{s_{rokgood}^{t+}}{y_{rokgood}^{t}}+\;\sum_{r=1}^{r_{2k}}\frac{s_{rokbad}^{t-}}{y_{rokbad}^{t}}+\sum_{k_{l}}^{ngood_{k}}\frac{s_{ok_{l}good}^{\left(t,t+1\right)}}{z_{ok_{l}good}^{\left(t,t+1\right)}})\right]}$$
  (b2) Division efficiency:
    (10) $$\phi_{0}^{*}=\min\frac{\sum_{t=1}^{T}W^{t}\left[1-\frac{1}{m_{k}+linkin_{k}+ninput_{k}}(\sum_{i=1}^{m_{k}}\frac{S_{iok}^{t-}}{x_{iok}^{t}}+\sum_{{\left(kh\right)}_{l}=1}^{linkin_{k}}\frac{s_{o{\left(kh\right)}_{l}in}^{t}}{z_{o{\left(kh\right)}_{l}in}^{t}}+\sum_{k_{l}}^{ninput_{k}}\frac{s_{ok_{l}input}^{\left(t,t+1\right)}}{z_{ok_{l}input}^{\left(t,t+1\right)}})\right]}{\sum_{t=1}^{T}W^{t}\;\left[1+\frac{1}{r_{1k}+r_{2k}}(\sum_{r=1}^{r_{1k}}\frac{s_{rokgood}^{t+}}{y_{rokgood}^{t}}+\;\sum_{r=1}^{r_{2k}}\frac{s_{rokbad}^{t-}}{y_{rokbad}^{t}})\right]}$$
  (b3) Division period efficiency:
    (11) $$\rho_{0}^{*}=\min\frac{1-\frac{1}{m_{k}+linkin_{k}+ninput_{k}}(\sum_{i=1}^{m_{k}}\frac{S_{iok}^{t-}}{x_{iok}^{t}}+\sum_{{\left(kh\right)}_{l}=1}^{linkin_{k}}\frac{s_{o{\left(kh\right)}_{l}in}^{t}}{z_{o{\left(kh\right)}_{l}in}^{t}}\sum_{k_{l}}^{ninput_{k}}\frac{s_{ok_{linput}input}^{\left(t,t+1\right)}}{z_{ok_{l}input}^{\left(t,t+1\right)}})}{1+\frac{1}{r_{1k}+r_{2k}}(\sum_{r=1}^{r_{1k}}\frac{s_{rokgood}^{t+}}{y_{rokgood}^{t}}+\;\sum_{r=1}^{r_{2k}}\frac{s_{rokbad}^{t-}}{y_{rokbad}^{t}})}$$

$\;x_{okgood}^{t}\;$: Labor, Energy consumed, Health expenditures
$y_{okgood}^{t}$: GDP, Birth rate
$y_{okbad}^{t}$: Phthisis, Death rate
$Z_{\left(kh\right)in}^{t}$: CO_2_, NO_X_, and SO_2_
$Z_{ok_{l}input}^{\left(t,\left(t+1\right)\right)}$: Fixed assets.

**Figure 2:**
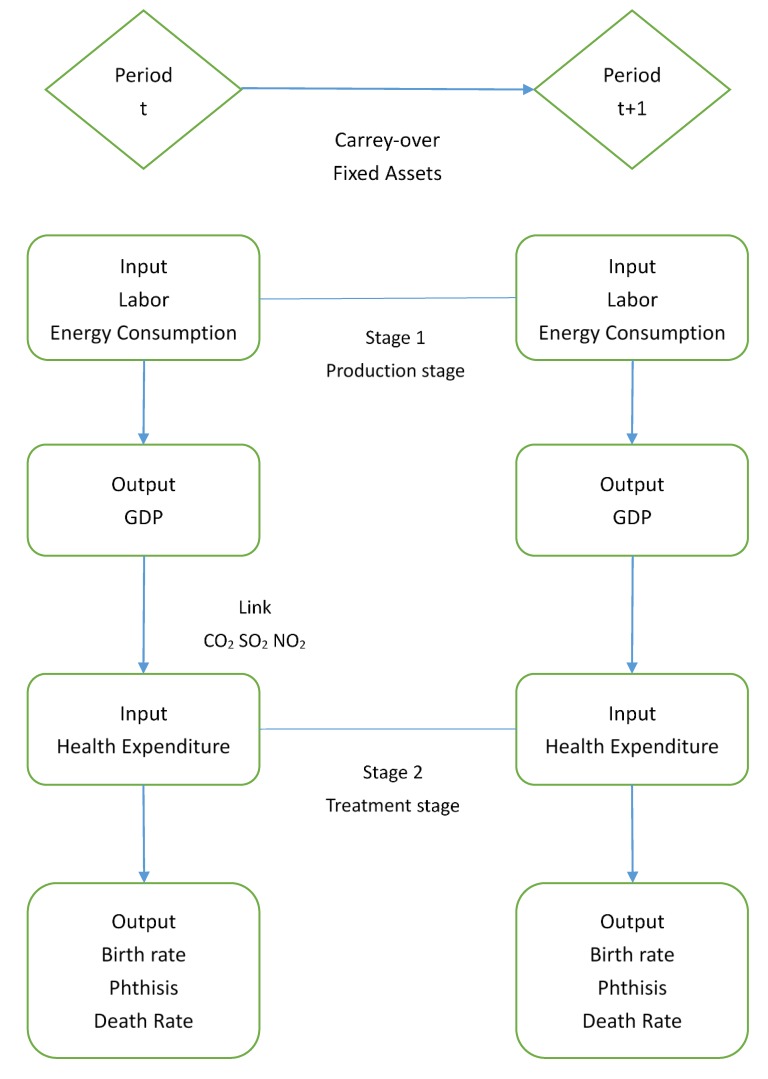
The structure of dynamic Network model.

Figure 2 reveals the framework of the modified undesirable dynamic network model of inter-temporal efficiency measurement and variables in this study.

### 3.4. Input and Output Efficiencies

We follow Hu and Wang’s [87] total-factor energy efficiency index to overcome any possible bias in the traditional energy efficiency indicator. There are seven key features of this present study: Energy consumed efficiency, CO_2_ efficiency, SO_2_ efficiency, NO_x_ efficiency, Health expenditure efficiency, Phthisis efficiency, and Death rate efficiency. In our study, “i” represents area and “t” represents time. The seven efficiency models are defined as follows.

(12) $$\mathrm{Energy}\;\mathrm{consumed}\;\mathrm{efficiency}\;=\;\frac{\mathrm{Target}\;\mathrm{Energy}\;\mathrm{consumed}\;\mathrm{Undesirable}\;\mathrm{output}\;\left(i,\;t\right)}{\mathrm{Actual}\;\mathrm{Energy}\;\mathrm{consumed}\;\mathrm{Undesirable}\;\mathrm{output}\;\left(i,\;t\right)}\;$$

(13) $${\mathrm{CO}}_{2}\;\mathrm{efficiency}\;=\;\frac{\mathrm{Target}\;{\mathrm{CO}}_{2}\;\mathrm{Undesirable}\;\mathrm{output}\;\left(i,\;t\right)}{\mathrm{Actual}\;{\mathrm{CO}}_{2}\;\mathrm{Undesirable}\;\mathrm{output}\;\left(i,\;t\right)}\;$$

(14) $${\mathrm{SO}}_{2}\mathrm{efficiency}\;=\;\frac{\mathrm{Target}\;{\mathrm{SO}}_{2}\;\mathrm{Undesirable}\;\mathrm{output}\;\left(i,\;t\right)}{\mathrm{Actual}\;{\mathrm{SO}}_{2}\;\mathrm{Undesirable}\;\mathrm{output}\;\left(i,\;t\right)}\;$$

(15) $${\mathrm{NO}}_{2}\;\mathrm{efficiency}\;=\;\frac{\mathrm{Target}\;{\mathrm{NO}}_{2}\;\mathrm{Undesirable}\;\mathrm{output}\;\left(i,\;t\right)}{\mathrm{Actual}\;{\mathrm{NO}}_{2}\;\mathrm{Undesirable}\;\mathrm{output}\;\left(i,\;t\right)}\;$$

(16) $$\mathrm{Health}\;\mathrm{Expenditure}\;\mathrm{efficiency}\;=\;\frac{\mathrm{Target}\;\mathrm{Health}\;\mathrm{Expenditure}\;\mathrm{input}\;\left(i,\;t\right)}{\mathrm{Actual}\;\mathrm{Health}\;\mathrm{Expenditure}\;\mathrm{input}\;\left(i,\;t\right)}\;$$

(17) $$\mathrm{Phthisis}\;\mathrm{efficiency}\;=\;\frac{\mathrm{Target}\;\mathrm{Phthisis}\;\mathrm{output}\;\left(i,\;t\right)}{\mathrm{Actual}\;\mathrm{Phthisis}\;\mathrm{output}\;\left(i,\;t\right)}\;$$

(18) $$\mathrm{Death}\;\mathrm{rate}\;\mathrm{efficiency}\;=\;\frac{\mathrm{Target}\;\mathrm{Death}\;\mathrm{rate}\;\mathrm{output}\;\left(i,\;t\right)}{\mathrm{Actual}\;\mathrm{Death}\;\mathrm{rate}\;\mathrm{output}\;\left(i,\;t\right)}\;$$

If the target Energy consumed and Health expenditure inputs equal the actual input, then the Energy consumed and health expenditure efficiencies equal 1, indicating overall efficiency. If the target Energy consumed and Health expenditure inputs are less than the actual input, then the Energy consumed and health expenditure efficiencies are less than 1, indicating overall inefficiency. If the target phthisis, death rate, CO_2_, SO_2_, and NO_2_ undesirable outputs equal the actual undesirable outputs, then phthisis, death rate, CO_2_, SO_2_ and NO_2_ efficiencies equal 1, indicating overall efficiency. If the target phthisis, death rate, CO_2_, SO_2_, and NO_2_ undesirable outputs are less than the actual undesirable outputs, then the phthisis, death rate, CO_2_, SO_2_ and NO_2_ efficiencies are less than 1, indicating overall inefficiency. From the above, we are able to obtain the overall efficiency, period efficiency, division efficiency, and division period efficiency for the 30 provinces for 2013–2016.

### 3.5. Data Sources and Description

This study uses panel data for economic and social developments from 2013 to 2016 in the 30 provinces. The data sources are [1], China Population and Employment Statistics Yearbook [88], China Health and Family Planning Statistical Yearbook [89], and China Energy Statistics Yearbook [90]. The 30 provinces have large differences in natural resources, total population, industrial structure, pollutant discharge, and governance.

The input and output variables are shown in Table 1. The inputs are labor and energy consumption, referring to [35,87,91]. The desirable output GDP refers to Wang et al. [92] and Li et al. [91]. For fixed assets as carry-over, we refer to Chang et al. [93]. The links Air, CO_2_, NO_x_, and SO_2_ are from Li et al. [94]. Input health expenditure, desirable output Birth rate, and undesirables Phthisis and Death rate come from Zhang [95]. The variables we employ are explained as follows.

## 4. Results and Discussion

### 4.1. Input-Output Index Statistical Analyses

**Figure 3:**
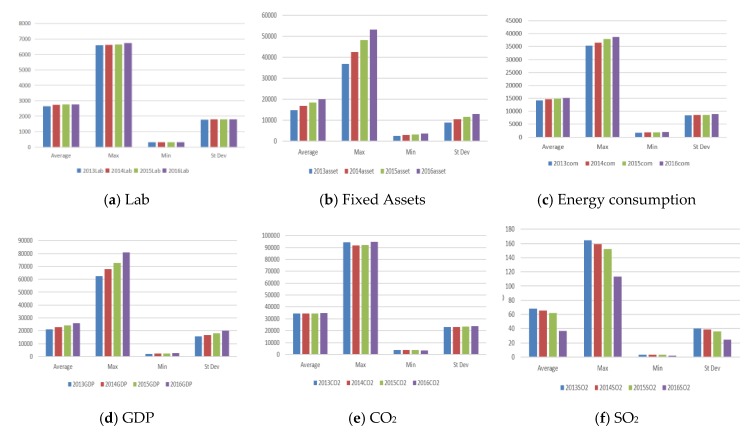

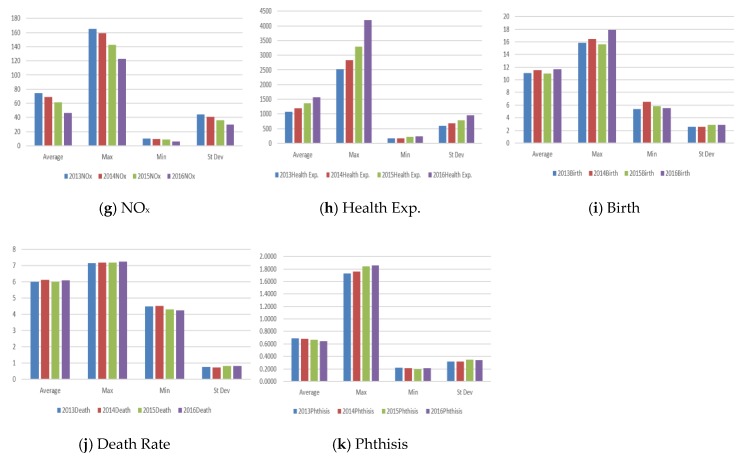
Statistical table of input and output indicators. Inputs include (**a**) Lab, (**c**) energy consumption, (**h**) Health Exp. Outputs include (**d**) GDP, (**i**) Birth, (**j**) Death Rate, (**k**) Phthisis. Carrey- over is (**b**) Fixed Assets. Link are (**e**) CO_2_, (**f**) SO_2_, (**g**) NO_X._

Figure 3 shows the statistics of input and output indicators. From labor and energy consumption, the maximum, minimum, and average values are all rising slowly over time. From fixed assets, the maximum, minimum, and average values show a slow rise in each year, and the rate of increase is larger than that of the two indicators noted above. For GDP, the maximum, minimum, and average values are rising slowly in each year.

For the CO_2_ indicator, the maximum, minimum, and average values of each year do not change much. Except for a slight decline in the 2015 average, the maximum value in 2014 has a downward trend, and the minimum value in 2016 shows a slight decrease. In other years, the values have slightly increased.

From the perspective of SO_2_ indicators, except for the slight increase in the minimum value in 2014, the maximum, minimum, and average values of other years show a downward trend. The downward trend in 2016 is the most obvious, denoting certain achievements occurred in energy conservation, emission reduction, and pollutant treatment.

From the NO_x_ index, the maximum, minimum, and average values of each year show a downward trend. The downward trend in 2016 is the most obvious. In recent years, there has been a downward trend in the emissions of atmospheric pollutants, indicating that there are certain effects in energy conservation, emission reduction, and pollutant treatment. From the second stage of health expenditures, the maximum, minimum, and average values are still rising slowly in each year, and the growth rate in 2016 is larger than the previous two years.

From the birth indicator, the maximum and average values for 2015 drop, and the maximum and average values for other years have an upward trend. The minimum shows a different trend, with the minimum in 2014 rising and falling in 2015 and 2016.

From the death indicator, the maximum and average values for 2015 decline, and the maximum and average values for other years show an upward trend. The minimum value rises in 2014 and then falls in 2015 and 2016.

From the phthisis indicator, there is a downward trend in the average of each year, and there is an upward trend in the maximum value of each year. The increase in 2015 is higher than that in other years. The minimum value drops slowly, but then increases in 2016.

### 4.2. Total Efficiency Scores for Each Year

Table 2 shows the overall efficiency scores of provinces from 2013 to 2016. Those with an overall efficiency score of 1 for all four years include Beijing, Tianjin, Shanghai, Hainan, Qinghai, and Ningxia. The efficiency score of Xinjiang in 2013 is higher than 0.8, but the efficiency score for the next three years is 1. There is room for improvement in the overall efficiency scores for each of the other provinces.

Provinces with an efficiency score above 0.5 include Jiangsu, Fujian, Shandong, and Guangdong. The efficiency scores of Fujian and Shandong are all lower than 0.6 in 2013, but then rise above 0.8 in the next three years, showing great efficiency improvement. The scores of Hebei, Shanxi, Inner Mongolia, Liaoning, Jilin, Heilongjiang, Zhejiang, Anhui, Jiangxi, Henan, Hubei, Hunan, Guangxi, Chongqing, Sichuan, Guizhou, Yunnan, Shaanxi, and Gansu are all below 0.5. There is room for improvement in the overall efficiency of these provinces.

The division of east, central, and west in the following table is based on the China Yearbook of Health Statistics. Eleven provinces in the eastern region include Beijing, Tianjin, Hebei, Liaoning, Shanghai, Jiangsu, Zhejiang, Fujian, Shandong, Guangdong, Hainan. Eight provinces in the central region include Heilongjiang, Jilin, Shanxi, Anhui, Jiangxi, Henan, Hubei, and Hunan. Twelve provinces in the western region include Inner Mongolia, Guangxi, Chongqing, Sichuan, Guizhou, Yunnan, Tibet, Shaanxi, Gansu, Qinghai, Ningxia, and Xinjiang.

The eastern region has the highest average efficiency, followed by the western region, and the lowest is in the central region. The average efficiency of the eastern region is about 0.7. The efficiencies of Beijing, Tianjin, Shanghai, Fujian, Shandong, and Hainan are mostly higher than the regional average efficiency and show less room for improvement. Hebei, Liaoning, Jiangsu, Zhejiang and Guangdong’s efficiency values in most years are below the regional average, and there is room for improvement. The average efficiency of the western region is less than 0.6. Qinghai, Ningxia, and Xinjiang’s efficiency values are 1. Most western provinces have efficiency values fluctuating around 0.4, and Sichuan has the lowest efficiency value. The average efficiency of the central region is below 0.4. Jiangxi has the highest efficiency. The lowest efficiency is in Heilongjiang at around 0.2, giving it large room for improvement.

Table 3 shows the correlation tests. The average efficiency value in 2013 is 0.5318, the average efficiency value in 2014 is 0.5716, the average efficiency value in 2015 is 0.5618, and the average efficiency value in 2016 is 0.5418. The average efficiency increases from 2013 to 2015, and decreases from 2015 to 2016. The correlation coefficient tests are all significant, and the efficiency values highly correlate over time.

### 4.3. Annual Efficiency Analysis at Each Stage

**Figure 4:**
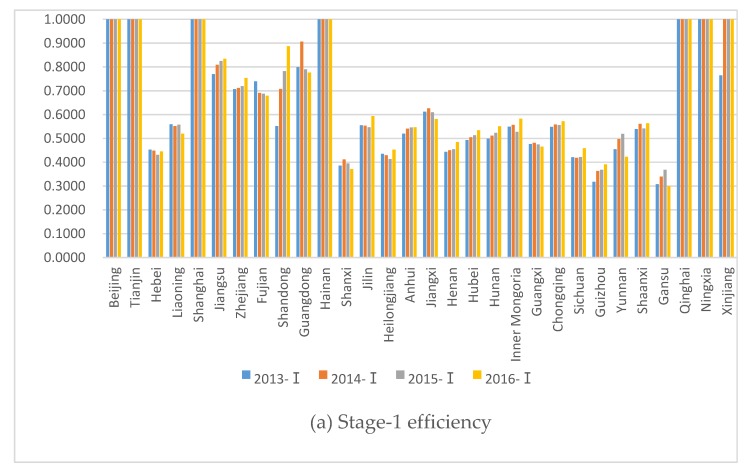

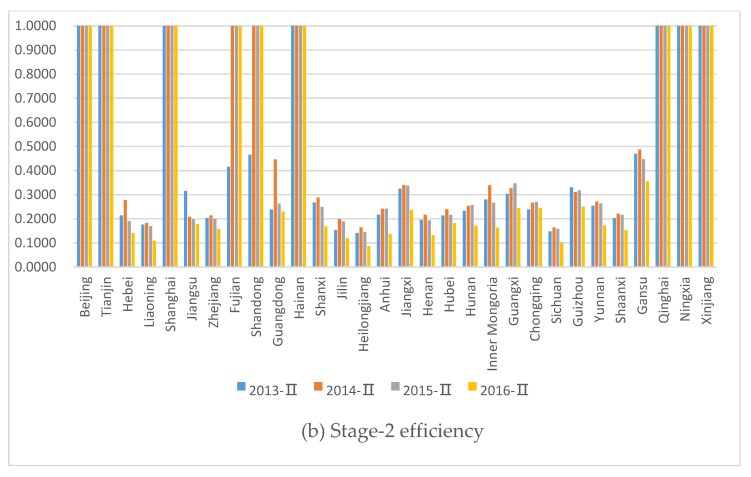
The efficiency of both stages by province during 2013–2016. (**a**) The efficiency of Stage 1. (**b**) The efficiency of Stage 2.

Table 4 and Figure 4 illustrate the two-stage efficiency scores for the 30 provinces from 2013 to 2016. Most provinces have better overall efficiency scores in the first stage than in the second stage.

From the first stage, Beijing, Tianjin, Shanghai, Hainan, Qinghai, and Ningxia have an efficiency score of 1 for all four years. Xinjiang’s efficiency score in 2013 is higher than 0.7 and is 1 in the last three years. Those with an efficiency below 1 and above 0.5 are Inner Mongolia, Liaoning, Jilin, Jiangsu, Zhejiang, Anhui, Fujian, Jiangxi, Shandong, Hubei, Hunan, Guangdong, Chongqing, and Shaanxi. Hebei, Shanxi, Heilongjiang, Henan, Guangxi, Yunnan, Gansu, Sichuan, and Guizhou are all lower than 0.5.

The eastern region has the highest average efficiency value in the first stage, followed by the western region, and the central region has the lowest average efficiency value. Beijing, Tianjin, Shanghai, and Hainan are higher than the eastern region’s average efficiency value, while Hebei, Hebei, and Liaoning are significantly lower than the eastern region’s average efficiency value. Shandong rises from below 0.6 in 2013 to nearly 0.9 in 2016. The average efficiency of the first stage in the western region is about 0.6. Efficiencies in Qinghai, Ningxia, and Xinjiang in most years are 1. The efficiency values of the other western provinces are all below 0.6, with Sichuan and Guizhou having the lowest efficiency values. The average efficiency of the first stage in the central region is around 0.5. Jiangxi has the highest efficiency value at above 0.6. The lowest efficiency in the central region is Shanxi at below 0.4, giving it much room for improvement.

From the second-stage health treatment efficiency score, the gap between the provinces is large. The provinces with a second-stage efficiency score of 1 include Beijing, Tianjin, Shanghai, Hainan, Qinghai, Ningxia, and Xinjiang. In 2013, the efficiency values of Fujian and Shandong are slightly higher than 0.4, but in the following three years they are 1. The provinces with efficiency values higher than 0.3 but less than 1 in each year are Guangxi, Guizhou, and Gansu. Jiangxi, Inner Mongolia, Jiangsu, and Guangdong are around 0.3. Hebei, Shanxi, Liaoning, Jilin, Heilongjiang, Zhejiang, Anhui, Henan, Hubei, Hunan, Chongqing, Sichuan, Yunnan, and Shaanxi all score below 0.3. The efficiency value of the second stage is much lower than the first stage in most provinces.

The eastern region has the highest average efficiency in the second stage, and the lowest is the central region. The average efficiency of the second stage for the eastern region fluctuates around 0.6, and the efficiencies of the provinces in the region present large difference. Beijing, Tianjin, Shanghai, and Hainan’s efficiency values are all 1. Hebei, Liaoning, Jiangsu, and Zhejiang’s efficiency values fluctuate around 0.2. The average efficiency value of the second stage in the western region is around 0.5. Qinghai, Ningxia, and Xinjiang have the best efficiency, and Sichuan has the lowest value. The average efficiency of the second stage in the central region is only about 0.2. The most efficient is Jiangxi with an efficiency value of less than 0.4. The lowest efficiency is in Heilongjiang, with annual efficiency below 0.2.

The provinces with the best efficiency values in the first and second stages are mostly concentrated in the eastern region. The western regions of Qinghai, Ningxia, and Xinjiang also perform well. The efficiency of the central region is at a relatively low level.

The two-stage efficiency values of Beijing, Tianjin, Shanghai, Hainan, Qinghai, Ningxia, and Xinjiang are all 1. The second-stage efficiency values of Fujian and Shandong are higher than their first-stage efficiency values. In most provinces, the efficiency value of the second stage is much lower than that of the first stage. For example, in the eastern provinces of Jiangsu, Zhejiang, and Guangdong, the efficiency values are higher than 0.7. The efficiency values for most years of the health treatment stage are 0.1 to 0.3. Thus, an improvement in economic development efficiency does not naturally lead to an improvement in health management efficiency. More measures need to be taken to improve the health efficiency of the relevant provinces.

From the first-stage efficiency values, those below 1 but are decreasing include Hebei, Shanxi, Liaoning, Fujian, Jiangxi, Guangxi, and Gansu, whereas most other provinces’ values below 1 are increasing. Shandong’s efficiency has increased the most. Guangdong’s efficiency increased significantly in 2014 and then declined in the next two years, going even lower in 2016 than the level in 2013.

The efficiency values in provinces that are below 1 show a downward trend. The health efficiency values of most provinces outside Jiangsu and Guizhou show an upward trend in 2014, but the efficiency values in 2015 and 2016 have a downward trend. Many provinces have improved their economic efficiency, but there is also a decline in the efficiency of health management.

Most provinces’ first-stage production efficiency has increased. Moreover, the efficiency value remains at a high level. However, the vast majority of provinces in the second stage have declining values. The health stage is thus inefficient and has much room for improvement.

Table 5 shows correlation tests, where from 2013 to 2016 the average efficiency of the first stage is between 0.6301 and 0.6589. The average efficiency of the second stage ranges from 0.4246 to 0.4887. The average efficiency value of the first stage is higher than the average efficiency value of the second stage. From 2013 to 2016, the correlation coefficient between the different year in first stage ranges from 0.9421 to 1.000, and the correlation coefficient between the different year in the second stage ranges from 0.9099 to 1.000. The correlation between the different year in first stage is high, but they are all highly correlated. The correlation between the efficiency value of the first stage and the second stage ranges from 0.7552 to 0.8192 and is also highly correlated.

### 4.4. Efficiency Scores and Rankings for Energy Consumption, CO_2_, SO_2_, and NO_x_ Efficiencies from 2013 to 2016

Table 6 shows the efficiency scores of energy consumption, CO_2_, SO_2_, and NO_x_ as input and output indicators for each province from 2013 to 2016.

From the energy consumption efficiency scores, we can see the gap between the provinces is large. The efficiency scores of Beijing, Tianjin, Shanghai, Hainan, Qinghai, and Ningxia are all 1 in the four years. The efficiency values of Fujian, Shandong, and Xinjiang in 2013 are above 0.8 and are all 1 in the other three years. Jilin, Jiangsu, Zhejiang, Anhui, Jiangxi, Henan, Hubei, Hunan, Guangdong, Guangxi, Chongqing, Yunnan, Shaanxi, and Gansu have efficiency scores below 1 and above 0.5. The provinces with energy consumption efficiency below 0.5 include Hebei, Shanxi, Inner Mongolia, Liaoning, Heilongjiang, Sichuan, and Guizhou. In provinces with efficiency below 1, the provinces with an increasing trend are Jilin, Zhejiang, Henan, Hubei, Hunan, and Sichuan. The provinces with a decreasing trend are Hebei, Shanxi, Inner Mongolia, Liaoning, Anhui, Jiangxi, Guangxi, Guangdong, Chongqing, Guizhou, Yunnan, and Shaanxi.

The eastern region has the best performance in energy consumption efficiency, with an average efficiency above 0.8. The lowest efficiency is in the central region at an average annual efficiency of about 0.6. Energy efficiency in most provinces is less than 0.5. Among them, Hebei and Liaoning belong to the eastern region, Shanxi and Heilongjiang belong to the central region, and Inner Mongolia, Sichuan, and Guizhou belong to the western region. There is much room for improvement in energy efficiency in these provinces.

From the CO_2_ efficiency score, the provinces with efficiency values of 1 in each year are Beijing, Tianjin, Shanghai, Hainan, Qinghai, Ningxia, and Xinjiang. Fujian and Shandong have efficiencies higher than 0.5 in 2013 and then at 1 in the next three years. The provinces with efficiency values less than 1 but greater than 0.5 are Jiangxi, Zhejiang, Hunan, Guangdong, Chongqing, Yunnan, and Gansu. Jiangxi, Zhejiang, Hunan, Guangdong, Chongqing, Yunnan, Gansu, Hebei, Shanxi, Inner Mongolia, Liaoning, Jilin, Heilongjiang, Jiangsu, Anhui, Henan, Hubei, Guangxi, Sichuan, Guizhou, and Shaanxi have efficiency values below 0.5.

The eastern region has the best CO_2_ efficiency performance with an average efficiency close to 0.8. The lowest average efficiency is in the central region, with an average annual efficiency at only about 0.6. Most provinces in the eastern region perform well, but Liaoning and Hebei have the lowest CO_2_ efficiency. Most provinces in the central region have poor CO_2_ efficiency. Jiangxi has the highest efficiency value at only about 0.6. Shanxi has the lowest efficiency value at around 0.2 in each year. Qinghai, Ningxia, and Xinjiang in the western region perform well. The lowest CO_2_ efficiency is in Inner Mongolia and Guizhou, which is around 0.3, and there is much room for improvement.

From the SO_2_ efficiency score, the provinces with efficiency values of 1 or nearly 1 in each year are Beijing, Tianjin, Shanghai, Hainan, Qinghai, Ningxia, Xinjiang, Fujian, and Guangdong. Shandong’s efficiency value in 2013 is only 0.4, but then hits 1 in the next 3 years. Provinces with an efficiency value less than 1 but greater than 0.5 are Jiangsu, Zhejiang, and Anhui. The efficiency values of Hebei, Shanxi, Inner Mongolia, Liaoning, Jilin, Heilongjiang, Jiangxi, Henan, Hubei, Hunan, Guangxi, Chongqing, Sichuan, Guizhou, Yunnan, Shaanxi, and Gansu are below 0.5.

The eastern region has the best SO_2_ efficiency performance with an average efficiency around 0.8. The lowest average efficiency is in the central region with an average annual efficiency below 0.4. Most provinces in the eastern region have better efficiency, but Liaoning and Hebei have the lowest SO_2_ efficiency value below 0.4. The efficiency value of Zhejiang in the first three years is only 0.6, and it increases to 1 in the fourth year. In the central region, Anhui has the highest efficiency value at around 0.7. Shanxi, Jilin and Heilongjiang have lower efficiency values. SO_2_ efficiency values in the central provinces are all increasing, with the largest increase in 2016. Qinghai, Ningxia, and Xinjiang perform well in the western region. Inner Mongolia, Guangxi, Sichuan, and Shaanxi’s efficiencies increase, while Chongqing and Yunnan’s efficiencies decrease. SO_2_ efficiency rises in most provinces.

From the NO_x_ efficiency score, the provinces with efficiency values of 1 each year are Beijing, Tianjin, Shanghai, Fujian, Hainan, Qinghai, Ningxia, and Xinjiang. Shandong’s efficiency in 2013 is only 0.5. Jiangsu, Zhejiang, Hunan, and Gansu have efficiency values less than one but greater than 0.5. Jiangxi’s efficiency value moves from 0.5 in the first three years to slightly higher than 0.3 in 2016. Yunnan’s efficiency value for the first three years goes from being above 0.7 to slightly higher than 0.3 in 2016. The efficiency values of Hebei, Shanxi, Inner Mongolia, Liaoning, Jilin, Heilongjiang, Anhui, Henan, Hubei, Guangdong, Guangxi, Chongqing, Sichuan, Guizhou, and Shaanxi are below 0.5.

The eastern region has the best NO_x_ efficiency performance, and the average efficiency is close to 0.8. The lowest average efficiency is in the central region, and the average annual efficiency is less than 0.4. Most provinces in the eastern region have better efficiency, but Hebei, Liaoning, Jiangsu, Zhejiang, and Guangdong are below the average efficiency value. In the central region, Jiangxi has the highest efficiency value with efficiency less than 0.6, and Heilongjiang has the lowest efficiency value at only 0.2. The average NO_x_ efficiency in the western region is about 0.6. Inner Mongolia has the worst efficiency performance with an efficiency value of only about 0.2 and has much room for improvement.

The provinces with higher energy consumption efficiency also have higher efficiency values of CO_2_, SO_2_, and NO_x_. Increasing energy consumption efficiency can reduce emissions of atmospheric pollutants.

Table 7 shows correlation tests, from 2013 to 2016, where the average Com efficiency value is 0.7037 to 0.7352, the average CO_2_ efficiency value is 0.5884 to 0.6384, the average SO_2_ efficiency value is 0.5113 to 0.6304, and the average NO_2_ efficiency value is 0.5613 to 0.6222. From 2013 to 2016, the Com correlation coefficient is 0.9648 to 1.000, the CO_2_ correlation coefficient is 0.9230 to 1.000, the SO_2_ correlation coefficient is 0.8724 to 1.000, and the NO_2_correlation coefficient is 0.9012 to 1.000. The correlations between Com and CO_2_, SO_2_, and NO_2_ are above 0.8, and the correlations between CO_2_ and SO_2_ and NO_2_ are also above 0.8. From the above results we see that the more energy consumption there is, the more air pollution arises from CO_2_, SO_2_, and NO_2_.

### 4.5. Efficiency Scores and Rankings for Health Expenditure, Death Rate, and Phthisis Efficiencies

Table 8 shows the efficiency scores of health expenditure, death rate, and phthisis input and output indicators for each province from 2013 to 2016.

From the health expenditure score, those with efficiency scores of 1 for four years include Beijing, Tianjin, Shanghai, Hainan, Qinghai, Ningxia, and Xinjiang. In Fujian and Shandong, their efficiency values are above 0.4 in 2013 and 1 for the other three years. Guizhou and Gansu have efficiency values above 0.3 and below 1. In Inner Mongolia, Guangxi, and Jiangxi, their efficiency values in the first three years are greater than 0.3, but fell below 0.3 in 2016. In 2013, Jiangsu’s efficiency value is higher than 0.3, but fell below 0.3 in the next three years. Guangdong’s efficiency value in 2014 is close to 0.5, but the other three years are below 0.3. The provinces with efficiency values below 0.3 are Hebei, Shanxi, Liaoning, Jilin, Heilongjiang, Zhejiang, Anhui, Henan, Hubei, Hunan, Chongqing, Sichuan, Yunnan, and Shaanxi. In 2014, Jiangsu and Guizhou show a downward trend. Hunan and Guangxi’s efficiency values for 2015 and 2016 are increasing. Health expenditure efficiency values are low, and the room for improvement is expanding.

The eastern region has the highest average health expenditure efficiency with an average efficiency around 0.6. The central region has the lowest average efficiency with an average efficiency of less than 0.3. Most provinces have efficiency values below 0.4 and have room for improvement.

For the death rate, those with efficiency score of 1 include Beijing, Tianjin, Shanghai, Hainan, Qinghai, Ningxia, and Xinjiang. Fujian’s 2013 efficiency value is above 0.8, and for the other three years it is 1. Those with efficiency values above 0.6 include Hebei, Shanxi, Zhejiang, Anhui, Jiangxi, Henan, Hubei, Hunan, Guangxi, Chongqing, Sichuan, Guizhou, Yunnan, Shaanxi, and Gansu. The efficiency value of Inner Mongolia is slightly higher than 0.6 in the first two years, but fell below 0.6 in the next two years. Jiangsu’s efficiency value in 2013 dropped from 0.8 to less than 0.6 in the next three years. The provinces with efficiency values below 0.6 are only Liaoning, Jilin, and Heilongjiang.

There is not much difference in the death rate among regions. The death rate efficiency of most provinces in the eastern region performs well, but the death rate efficiencies of Liaoning and Jiangsu are relatively low. There is not much difference in the efficiency of the death rate among the provinces in the central region. Anhui, Jiangxi, and Hunan have the highest efficiency, and Jilin and Heilongjiang have the lowest efficiency. The efficiency values of the provinces in the western region exhibit less difference. Qinghai, Ningxia, and Xinjiang have an efficiency value of 1, while Inner Mongolia, Chongqing, and Sichuan have relatively low efficiency values around 0.6.

In the 20 provinces with efficiency below 1, most provinces show a downward worsening trend. Hebei, Heilongjiang, and Jiangsu present the most distinct declining trend. Those with an upward trend are Jiangxi, Guangxi, Chongqing, and Guizhou.

For phthisis, those with an efficiency score of 1 for four years include Beijing, Tianjin, Shanghai, Fujian, Shandong, Hainan, Gansu, Qinghai, Ningxia, and Xinjiang. The efficiency values of Anhui, Jiangxi, Yunnan, and Shaanxi are all 1 in the first three years, but decline in 2016. The efficiency value of Guangdong in the first two years is about 0.6 and in the last two years is 1. For the first two years, Hebei has an efficiency value of 1, but in the next two years it fell to above 0.8. Those provinces with efficiency values below 1 and greater than 0.7 are Hebei, Shanxi, Jiangsu, Zhejiang, Inner Mongolia, Henan, Hubei, Hunan, Guangxi, Chongqing, and Sichuan. Provinces with efficiency values below 0.7 are Liaoning, Jilin, Heilongjiang, and Guizhou. Most provinces show high efficiency at controlling phthisis.

The average efficiency of phthisis in the western region is leading the other two regions in the first three years, while the average efficiency of phthisis in the eastern region is leading in the fourth year. The worst performance in the eastern region is Liaoning. In the eastern region, the efficiency of phthisis in Guangdong increases, while Hebei, Liaoning, Jiangsu, and Zhejiang show a downward trend. The lowest efficiency of phthisis in the central region is in Jilin and Heilongjiang. Except for the increase in efficiency in Hubei, the efficiency in other central provinces declines. Most provinces in the western region have higher phthisis efficiency, and Guizhou has the lowest efficiency value at about 0.6. Except for Chongqing having a rising efficiency, most other western provinces show a downward trend in efficiency. From the above analysis, we see that although the efficiency of phthisis governance in many provinces is at a high level, the efficiency declines in many provinces.

Table 9 shows correlation tests, whereby from 2013 to 2016 the average health efficiency value is 0.4448 to 0.5117, the average death rate efficiency is 0.7811 to 0.8029, and the average efficiency of phthisis is 0.8223 to 0.9297. For 2013 to 2016, the health correlation coefficient is 0.9051 to 1.000, the death correlation coefficient is 0.9364 to 1.000, and the phthisis correlation coefficient is 0.6862 to 1.000. The correlation between health expenditure and death is higher than between phthisis, indicating that health expenditure has a positive relationship with lower mortality. Health expenditure, death, and phthisis are all highly correlated.

Table 10 summarizes the efficiency of health treatment stages in various regions, which can help us compare the efficiency characteristics of variables in different places.

## 5. Conclusions

This study uses the Modified Undesirable Dynamic Two Stage DEA model to assess productivity and health management efficiencies in 30 provinces of China from 2013 to 2016. Variables include labor, fixed assets, energy consumption, GDP, CO_2_, SO_2_, NO_x_, health expenditure, birth rate, death rate, and tuberculosis incidence. The following conclusions are drawn from the findings here.

(1) From the average, maximum, and minimum values, we see that labor, fixed assets, and energy consumption inputs and GDP output in the production stage are rising from 2013 to 2016. Atmospheric pollutants SO_2_ and NO_x_ show a downward trend, with the most distinct declining trend in 2016. From the analysis of the health governance stage, the amount of health expenditure is also increasing year by year, but the changes in birth rate, death rate, and tuberculosis incidence are not large.

(2) The overall efficiencies of Beijing, Tianjin, Shanghai, Hainan, Qinghai, and Ningxia hit 1 for all years. Xinjiang’s efficiency score in 2013 is higher than 0.8 and then moves to 1 in the next three years. In provinces with overall efficiency value below 1, the efficiency values of 19 provinces are all below 0.5. From a regional comparison, the highest average overall efficiency is in the eastern region, second is the western region, and the lowest is the central region. The average overall efficiency in the eastern region is about 0.7, while the average overall efficiency in the western region is only about 0.4. 

(3) From the first stage, Beijing, Tianjin, Shanghai, Hainan, Qinghai, and Ningxia all have a production efficiency score of 1 for four consecutive years. Xinjiang’s efficiency score in 2013 is higher than 0.7, and its total efficiency is 1 in the last three years. The annual efficiency values of Hebei, Shanxi, Heilongjiang, Henan, Guangxi, Yunnan, Gansu, Sichuan, and Guizhou are below 0.5. From a regional comparison, the highest average efficiency of the first stage is in the eastern region, second is the western region, and lowest is the central region. The average efficiency in the eastern region is about 0.8, and the average efficiency in the central region is around 0.5.

(4) The efficiencies in the second stage for most provinces are much lower than those in the first stage. The provinces with an efficiency score of 1 in the second stage include Beijing, Tianjin, Shanghai, Hainan, Qinghai, Ningxia, and Xinjiang. In 2013, the efficiency values of Fujian and Shandong are slightly higher than 0.4 and then turn to 1 in the following three years. Provinces with an efficiency value higher than 0.3 but lower than 1 are Guangxi, Guizhou, and Gansu. In the first three years of Jiangxi, its efficiency value is higher than 0.3, but then fell below 0.3 in 2016. The efficiency values of the other 17 provinces are below 0.3. The second stage clearly has room for efficiency improvement. From a regional comparison, the average efficiency of the second stage of the eastern region is the highest, and the lowest is the central region. The efficiency values of the provinces in the region exhibit large differences.

(5) The two-stage efficiency values of Beijing, Tianjin, Shanghai, Hainan, Qinghai, Ningxia, and Xinjiang are equal to or close to 1. The second-stage efficiency values of Fujian and Shandong are higher than their first-stage efficiency values. In most other provinces, their second-stage efficiency values are much lower than the first stage. The efficiency values in the production stages of Jiangsu, Zhejiang, and Guangdong are higher than 0.7, but the efficiency values for most years of the health treatment stage are low at between 0.1 and 0.3.

(6) Among the provinces with annual efficiency values below 1, most of them have a downward trend in energy consumption, CO_2_, and NO_x_ efficiency values in the four years. However, their SO_2_ efficiency values are on the rise, and the growth rates of SO_2_ efficiency in Liaoning, Jilin, Heilongjiang, Zhejiang, Anhui, Henan, Hubei, Hunan, Sichuan, and Shaanxi are much higher than in previous years. In the provinces with higher energy consumption efficiency, the efficiencies of CO_2_, SO_2_, and NO_x_ are also high. The eastern region has the highest average efficiency of these variables, while the central region has the worst efficiency performance.

(7) Among the provinces with health expenditure efficiencies below 1, the efficiency values of most provinces are around 0.3, with trending declines in the next three years. From a regional comparison, the eastern region has the highest average health expenditure efficiency with an average efficiency around 0.6, and the central region has the lowest average efficiency with an average efficiency of less than 0.3.

(8) For the death rate, most provinces have higher efficiency values. Beijing, Tianjin, Shanghai, Shandong, Guangdong, Hainan, Qinghai, Ningxia, and Xinjiang have efficiency scores of 1 in four years. The efficiencies of Hebei, Heilongjiang, and Jiangsu are declining, while the efficiencies of Jiangxi, Guangxi, Chongqing, and Guizhou are increasing. With economic development and the improvement of people’s living standards, most provinces have achieved a higher level of death management efficiency. From a regional comparison, the average death rate efficiency in the eastern region is slightly better, and there is not much difference between regions.

(9) For phthisis, those with efficiency scores of 1 for four years include Beijing, Tianjin, Shanghai, Fujian, Shandong, Hainan, Gansu, Qinghai, Ningxia, and Xinjiang. Those with efficiencies around 0.5 are Liaoning, Jilin, Heilongjiang, and Guizhou. Most provinces exhibit high efficiency in the control of tuberculosis infections. From a regional comparison, the average efficiency of phthisis in the western region is slightly ahead in the first three years, and the eastern region is slightly ahead in the fourth year. Many provinces show a downward trend in phthisis efficiency.

### Policy Suggestion

(1) Increasing the efficiency of energy consumption can effectively reduce emissions of greenhouse gases and atmospheric pollutants such as sulfur dioxide and nitrogen oxides. There are many measures to improve energy consumption efficiency, such as continuing to eliminate high energy consumption, improving the low efficiency of backward production capacity, strengthening publicity to raise public awareness of energy conservation and environmental protection, advocating green lifestyles represented by green behavior, and developing clean energy with less environmental pollution.

(2) From the rapid improvement of sulfur dioxide emission efficiency in most provinces, it can be seen that technology applications such as desulfurization and sulfur reduction in the production process advocated by the government in recent years have achieved great results. The government should promote broader practical applications of environmental protection technologies through administrative purchases, tax incentives, media publicity, and other means to improve the emission efficiency of atmospheric pollutants such as greenhouse gases and nitrogen oxides.

(3) There are two reasons for the inefficiency of health expenditures. First, more money is spent on disease treatment than on prevention, and the government is helping to increase the efficiency of health expenditures through advocacy and disease prevention. Second, the emission of pollutants and the deterioration of the environment make health problems very serious. Therefore, the government has adopted measures such as health promotion, disease prevention, and environmental pollution control measures to help improve the efficiency of health spending.

(4) In Liaoning, Jilin, Heilongjiang, and other provinces, CO_2_, NO_x_, health expenditure, mortality, and phthisis are relatively inefficient. The government needs to adjust these areas’ industrial structure, environmental pollution rectification, disease prevention, and disease treatment.

## Figures and Tables

**Table 1:** Input and output variables of two stages.

Variable			Explanation
Stage I	Input	Labor (Lab)	The number of labor workers in each province at year end. Unit: 10,000
		Energy consumed (Com)	Calculated from the total energy consumption in each province. Unit: 10,000 tons of standard coal
	Output	GDP	The final results of the production activities of the resident units in each place of that year. Unit: 100 million CNY
Link		CO_2_	Carbon dioxide and greenhouse gases. Unit: 10,000 tons of standard coal
		SO_2_	Sulfur dioxide emissions. Unit: 10,000 tons of standard coal
		NOx	Emissions of nitrogen oxide in the air, including a variety of compounds, such as NO_2_, N_2_O, NO, N_2_O_3_, N_2_O_3_. Unit: 10,000 tons of standard coal
Stage II	Input	Health expenditures (Health exp.)	Total health expenditures for whole year. Unit: 100 million RMB
	Output	Birth rate (Birth)	Ratio of the number of births in each province to the average number of births in the same period. Unit: ‰
		Phthisis	Incidence of Phthisis infectious diseases in the population. Unit: ‰
		Death rate (Death)	Ratio of deaths of each province to the average number of deaths in the same period. Unit: ‰
Carry-over		Fixed assets (Asset)	Capital stock in each province. Unit: 100 million CNY

**Table 2:** Overall efficiency of each province.

DMU	Region	2013	2014	2015	2016
Beijing	East	1.0000	1.0000	1.0000	1.0000
Tianjin	East	1.0000	1.0000	1.0000	1.0000
Hebei	East	0.3337	0.3630	0.3112	0.2930
Liaoning	East	0.3678	0.3679	0.3637	0.3148
Shanghai	East	1.0000	1.0000	1.0000	1.0000
Jiangsu	East	0.5426	0.5084	0.5112	0.5063
Zhejiang	East	0.4547	0.4628	0.4596	0.4563
Fujian	East	0.5775	0.8456	0.8437	0.8397
Shandong	East	0.5084	0.8539	0.8910	0.9436
Guangdong	East	0.5189	0.6761	0.5259	0.5028
Hainan	East	1.0000	1.0000	1.0000	1.0000
Shanxi	Central	0.3267	0.3503	0.3220	0.2698
Jilin	Central	0.3543	0.3763	0.3680	0.3567
Heilongjiang	Central	0.2880	0.2971	0.2786	0.2702
Anhui	Central	0.3687	0.3917	0.3942	0.3415
Jiangxi	Central	0.4686	0.4834	0.4734	0.4092
Henan	Central	0.3198	0.3337	0.3239	0.3083
Hubei	Central	0.3540	0.3725	0.3650	0.3582
Hunan	Central	0.3662	0.3828	0.3902	0.3615
Inner Mongoria	West	0.4148	0.4481	0.3975	0.3730
Guangxi	West	0.3901	0.4038	0.4114	0.3549
Chongqing	West	0.3938	0.4124	0.4135	0.4081
Sichuan	West	0.2846	0.2915	0.2899	0.2802
Guizhou	West	0.3243	0.3372	0.3432	0.3209
Yunnan	West	0.3545	0.3843	0.3916	0.2978
Shaanxi	West	0.3711	0.3917	0.3789	0.3584
Gansu	West	0.3888	0.4131	0.4077	0.3277
Qinghai	West	1.0000	1.0000	1.0000	1.0000
Ningxia	West	1.0000	1.0000	1.0000	1.0000
Xinjiang	West	0.8823	1.0000	1.0000	1.0000
Average Efficiency	East	0.6640	0.7343	0.7188	0.7142
	Central	0.3558	0.3735	0.3644	0.3344
	West	0.5277	0.5529	0.5485	0.5201

**Table 3:** Correlation test of efficiency values from 2013 to 2016.

Year	2013	2014	2015	2016
2013	1.000	0.9553	0.9524	0.9419
2014	0.9553	1.000	0.9938	0.9901
2015	0.9524	0.9938	1.0000	0.9965
2016	0.9419	0.9901	0.9965	1.0000
Average	0.5318	0.5716	0.5618	0.5418

**Table 4:** Two-stage total efficiency score during 2013–2016.

DMU	Region	2013—I	2013—II	2014—I	2014—II	2015—I	2015—II	2016—I	2016—II
Beijing	East	1.0000	1.0000	1.0000	1.0000	1.0000	1.0000	1.0000	1.0000
Tianjin	East	1.0000	1.0000	1.0000	1.0000	1.0000	1.0000	1.0000	1.0000
Hebei	East	0.4530	0.2143	0.4484	0.2776	0.4313	0.1911	0.4452	0.1408
Liaoning	East	0.5598	0.1758	0.5523	0.1835	0.5580	0.1694	0.5203	0.1092
Shanghai	East	1.0000	1.0000	1.0000	1.0000	1.0000	1.0000	1.0000	1.0000
Jiangsu	East	0.7692	0.3159	0.8089	0.2080	0.8246	0.1979	0.8339	0.1786
Zhejiang	East	0.7064	0.2030	0.7119	0.2137	0.7194	0.1998	0.7540	0.1586
Fujian	East	0.7391	0.4159	0.6912	1.0000	0.6873	1.0000	0.6793	1.0000
Shandong	East	0.5518	0.4650	0.7078	1.0000	0.7820	1.0000	0.8872	1.0000
Guangdong	East	0.7990	0.2388	0.9059	0.4463	0.7893	0.2626	0.7761	0.2296
Hainan	East	1.0000	1.0000	1.0000	1.0000	1.0000	1.0000	1.0000	1.0000
Shanxi	Central	0.3855	0.2678	0.4117	0.2889	0.3948	0.2493	0.3714	0.1682
Jilin	Central	0.5552	0.1534	0.5538	0.1989	0.5472	0.1889	0.5938	0.1197
Heilongjiang	Central	0.4351	0.1409	0.4295	0.1646	0.4132	0.1441	0.4532	0.0871
Anhui	Central	0.5200	0.2173	0.5412	0.2422	0.5459	0.2424	0.5469	0.1362
Jiangxi	Central	0.6125	0.3247	0.6268	0.3401	0.6095	0.3373	0.5814	0.2371
Henan	Central	0.4437	0.1960	0.4507	0.2166	0.4548	0.1929	0.4845	0.1321
Hubei	Central	0.4935	0.2144	0.5052	0.2398	0.5135	0.2164	0.5347	0.1818
Hunan	Central	0.4994	0.2330	0.5121	0.2536	0.5235	0.2568	0.5510	0.1721
Inner Mongoria	West	0.5491	0.2806	0.5571	0.3390	0.5277	0.2673	0.5827	0.1633
Guangxi	West	0.4767	0.3034	0.4814	0.3263	0.4752	0.3476	0.4653	0.2446
Chongqing	West	0.5485	0.2390	0.5582	0.2667	0.5563	0.2707	0.5719	0.2443
Sichuan	West	0.4212	0.1481	0.4187	0.1643	0.4216	0.1583	0.4585	0.1018
Guizhou	West	0.3176	0.3311	0.3636	0.3109	0.3680	0.3183	0.3913	0.2505
Yunnan	West	0.4545	0.2546	0.4971	0.2716	0.5196	0.2636	0.4232	0.1724
Shaanxi	West	0.5391	0.2032	0.5615	0.2218	0.5415	0.2163	0.5639	0.1528
Gansu	West	0.3080	0.4695	0.3395	0.4867	0.3687	0.4467	0.2995	0.3560
Qinghai	West	1.0000	1.0000	1.0000	1.0000	1.0000	1.0000	1.0000	1.0000
Ningxia	West	1.0000	1.0000	1.0000	1.0000	1.0000	1.0000	1.0000	1.0000
Xinjiang	West	0.7646	1.0000	1.0000	1.0000	1.0000	1.0000	1.0000	1.0000
Average Efficiency	East	0.7799	0.5481	0.8024	0.6663	0.7993	0.6382	0.8087	0.6197
	Central	0.4931	0.2184	0.5039	0.2431	0.5003	0.2285	0.5146	0.1543
	West	0.5799	0.4754	0.6161	0.4898	0.6162	0.4808	0.6142	0.4260

**Table 5:** Correlation test of efficiency value in two stages from 2013 to 2016.

	2013—I	2013—II	2014—I	2014—II	2015—I	2015—II	2016—I	2016—II
2013—I	1.0000	0.8192	0.9718	0.7671	0.9617	0.7552	0.9421	0.7726
2013—II	0.8192	1.0000	0.8378	0.9099	0.8607	0.9143	0.8265	0.9129
Average	0.6301	0.4335	0.6545	0.4887	0.6524	0.4713	0.6589	0.4246

**Table 6:** Annual energy consumption, CO_2_, SO_2_, and NO_x_ efficiencies.

DMU	Region	2013 Com	2014 Com	2015 Com	2016 Com	2013 CO_2_	2014 CO_2_	2015 CO_2_	2016 CO_2_	2013 SO_2_	2014 SO_2_	2015 SO_2_	2016 SO_2_	2013 NO_x_	2014 NO_x_	2015 NO_x_	2016 NO_x_
Beijing	East	1.0000	1.0000	1.0000	1.0000	1.0000	1.0000	1.0000	1.0000	1.0000	1.0000	1.0000	1.0000	1.0000	1.0000	1.0000	1.0000
Tianjin	East	1.0000	1.0000	1.0000	1.0000	1.0000	1.0000	1.0000	1.0000	1.0000	1.0000	1.0000	1.0000	1.0000	1.0000	1.0000	1.0000
Hebei	East	0.4380	0.4340	0.4049	0.3903	0.2569	0.2600	0.2343	0.2005	0.3688	0.3747	0.3441	0.3268	0.3430	0.3401	0.3113	0.2117
Liaoning	East	0.4526	0.4520	0.4324	0.3913	0.2722	0.2670	0.2475	0.2327	0.1958	0.1913	0.1785	0.3179	0.2882	0.2865	0.2638	0.2583
Shanghai	East	1.0000	1.0000	1.0000	1.0000	1.0000	1.0000	1.0000	1.0000	1.0000	1.0000	1.0000	1.0000	1.0000	1.0000	1.0000	1.0000
Jiangsu	East	0.7426	0.7942	0.7880	0.7700	0.4265	0.4995	0.4741	0.4566	0.4789	0.6190	0.6181	0.6362	0.4480	0.5553	0.5623	0.4606
Zhejiang	East	0.7510	0.7597	0.7473	0.7573	0.5635	0.5957	0.5791	0.6165	0.5550	0.5823	0.6082	1.0000	0.5735	0.6205	0.6413	0.7781
Fujian	East	0.9214	1.0000	1.0000	1.0000	0.8132	1.0000	1.0000	1.0000	0.9789	1.0000	1.0000	1.0000	1.0000	1.0000	1.0000	1.0000
Shandong	East	0.8984	1.0000	1.0000	1.0000	0.5604	1.0000	1.0000	1.0000	0.4056	1.0000	1.0000	1.0000	0.5329	1.0000	1.0000	1.0000
Guangdong	East	0.9177	0.9623	0.8599	0.7996	0.8837	0.9525	0.8070	0.6588	0.9170	1.0000	0.9247	1.0000	0.7253	0.8507	0.7011	0.5563
Hainan	East	1.0000	1.0000	1.0000	1.0000	1.0000	1.0000	1.0000	1.0000	1.0000	1.0000	1.0000	1.0000	1.0000	1.0000	1.0000	1.0000
Shanxi	Central	0.3089	0.3553	0.3355	0.3019	0.2248	0.2902	0.2743	0.2038	0.1900	0.2671	0.2327	0.2378	0.2601	0.3420	0.3077	0.2072
Jilin	Central	0.5525	0.5680	0.5627	0.6057	0.2973	0.3042	0.2889	0.3469	0.2539	0.2511	0.2126	0.5061	0.2543	0.2547	0.2229	0.3176
Heilongjiang	Central	0.4485	0.4437	0.4060	0.4375	0.3064	0.2886	0.2534	0.3059	0.2197	0.2171	0.2024	0.3774	0.2154	0.2100	0.1942	0.2122
Anhui	Central	0.7248	0.7694	0.7733	0.7107	0.4386	0.4840	0.4879	0.4131	0.5944	0.6723	0.6657	0.8567	0.4391	0.4906	0.5033	0.4052
Jiangxi	Central	0.8530	0.8850	0.8444	0.7104	0.5974	0.6656	0.6173	0.4298	0.3818	0.4628	0.4114	0.4501	0.5091	0.5731	0.5441	0.3144
Henan	Central	0.5548	0.5703	0.5723	0.5977	0.3729	0.4080	0.4140	0.4371	0.2533	0.2851	0.2879	0.7187	0.2680	0.3041	0.3104	0.3487
Hubei	Central	0.5688	0.5842	0.5767	0.5857	0.4196	0.4513	0.4204	0.4463	0.3036	0.3131	0.2851	0.4656	0.4423	0.4644	0.4348	0.4350
Hunan	Central	0.6052	0.6306	0.6389	0.6393	0.4965	0.5432	0.5177	0.4992	0.2811	0.3223	0.3286	0.5179	0.4758	0.5150	0.5400	0.4556
Inner Mongoria	West	0.3581	0.3960	0.3369	0.3403	0.1611	0.2020	0.1615	0.1792	0.1041	0.1621	0.1215	0.2753	0.1509	0.2134	0.1649	0.2295
Guangxi	West	0.6283	0.6393	0.6183	0.5522	0.4627	0.5002	0.5035	0.3651	0.2916	0.3301	0.3033	0.3496	0.4128	0.4783	0.4813	0.3428
Chongqing	West	0.6105	0.6237	0.6048	0.5688	0.5719	0.5763	0.5542	0.4495	0.2308	0.2468	0.2104	0.1922	0.4786	0.4996	0.4796	0.4191
Sichuan	West	0.4979	0.4915	0.4918	0.5232	0.4344	0.4508	0.4677	0.5746	0.2463	0.2343	0.2495	0.4041	0.4598	0.4577	0.4597	0.4547
Guizhou	West	0.4463	0.4146	0.4165	0.3863	0.3011	0.3175	0.2965	0.2163	0.1233	0.1515	0.1380	0.1016	0.3028	0.3429	0.3391	0.2350
Yunnan	West	0.6770	0.6902	0.7274	0.5924	0.6914	0.8200	0.9420	0.5947	0.4589	0.5345	0.5450	0.2996	0.7114	0.7777	0.7783	0.3265
Shaanxi	West	0.5749	0.6200	0.5803	0.5417	0.3662	0.4371	0.4245	0.4008	0.1660	0.2380	0.2327	0.4387	0.2682	0.3495	0.3454	0.3598
Gansu	West	0.9443	0.9728	1.0000	0.9081	0.7334	0.8378	0.9547	0.6604	0.3389	0.4309	0.5116	0.4410	0.6072	0.7396	0.8438	0.5120
Qinghai	West	1.0000	1.0000	1.0000	1.0000	1.0000	1.0000	1.0000	1.0000	1.0000	1.0000	1.0000	1.0000	1.0000	1.0000	1.0000	1.0000
Ningxia	West	1.0000	1.0000	1.0000	1.0000	1.0000	1.0000	1.0000	1.0000	1.0000	1.0000	1.0000	1.0000	1.0000	1.0000	1.0000	1.0000
Xinjiang	West	0.8207	1.0000	1.0000	1.0000	1.0000	1.0000	1.0000	1.0000	1.0000	1.0000	1.0000	1.0000	1.0000	1.0000	1.0000	1.0000
Average Efficiency	East	0.8293	0.8547	0.8393	0.8281	0.7069	0.7795	0.7584	0.7423	0.7182	0.7970	0.7885	0.8437	0.7192	0.7866	0.7709	0.7514
	Central	0.5771	0.6008	0.5887	0.5736	0.3942	0.4294	0.4092	0.3853	0.3097	0.3489	0.3283	0.5163	0.3580	0.3942	0.3822	0.3370
	West	0.6871	0.7135	0.7069	0.6739	0.6111	0.6492	0.6640	0.5855	0.4509	0.4844	0.4829	0.5002	0.5811	0.6235	0.6265	0.5345

**Table 7:** Com, CO_2_, SO_2_, and NO_2_ Efficiency Correlation Tests from 2013 to 2016.

	2013 Com	2014 Com	2015 Com	2016 Com	2013 CO_2_	2014 CO_2_	2015 CO_2_	2016 CO_2_	2013 SO_2_	2014 SO_2_	2015 SO_2_	2016 SO_2_	2013 NO_2_	2014 NO_2_	2015 NO_2_	2016 NO_2_
2013Com	1.0000	0.9855	0.9821	0.9648	0.9199	0.9402	0.9246	0.8969	0.8560	0.8933	0.9050	0.8220	0.8831	0.9206	0.9219	0.8574
2014Com	0.9855	1.0000	0.9943	0.9765	0.9065	0.9489	0.9311	0.9044	0.8518	0.9110	0.9214	0.8402	0.8727	0.9298	0.9292	0.8665
2015Com	0.9821	0.9943	1.0000	0.9832	0.9059	0.9450	0.9449	0.9159	0.8413	0.8995	0.9156	0.8269	0.8812	0.9352	0.9448	0.8737
2016Com	0.9648	0.9768	0.9832	1.0000	0.9002	0.9346	0.9233	0.9496	0.8627	0.9128	0.9297	0.8818	0.8859	0.9287	0.9382	0.9282
2013 CO_2_	0.9199	0.9065	0.9059	0.9002	1.0000	0.9562	0.9410	0.9230	0.9216	0.8842	0.8922	0.7696	0.9738	0.9436	0.9335	0.8727
2014 CO_2_	0.9402	0.9489	0.9499	0.9346	0.9562	1.0000	0.9896	0.9539	0.8670	0.9176	0.9276	0.7882	0.9360	0.9859	0.9775	0.8935
2015 CO_2_	0.9246	0.9311	0.9449	0.9233	0.9410	0.9896	1.0000	0.9472	0.8306	0.8812	0.8999	0.7436	0.9264	0.9757	0.9814	0.8741
2016 CO_2_	0.8969	0.9044	0.9159	0.9496	0.9230	0.9539	0.9472	1.0000	0.8746	0.9120	0.9285	0.8529	0.9316	0.9599	0.9621	0.9716
2013 SO_2_	0.8560	0.8518	0.8413	0.8627	0.9216	0.8670	0.8306	0.8746	1.0000	0.9475	0.9438	0.8724	0.9471	0.8929	0.8663	0.8749
2014 SO_2_	0.8933	0.9111	0.8995	0.9128	0.8842	0.9177	0.8812	0.9120	0.9475	1.0000	0.9968	0.9102	0.9017	0.9434	0.9171	0.9082
2015 SO_2_	0.9050	0.9214	0.9160	0.9297	0.8922	0.9276	0.8999	0.9285	0.9438	0.9968	1.0000	0.9132	0.9108	0.9535	0.9359	0.9219
2016 SO_2_	0.8220	0.8402	0.8269	0.8818	0.7696	0.7882	0.7436	0.8529	0.8724	0.9102	0.9132	1.0000	0.7736	0.8022	0.7849	0.8862
2013 NO_2_	0.8831	0.8727	0.8812	0.8859	0.9738	0.9360	0.9264	0.9316	0.9471	0.9019	0.9108	0.7736	1.0000	0.9544	0.9472	0.9015
2014 NO_2_	0.9206	0.9298	0.9352	0.9289	0.9436	0.9859	0.9757	0.9599	0.8929	0.9434	0.9535	0.8022	0.9544	1.0000	0.9923	0.9235
2015 NO_2_	0.9219	0.9292	0.9448	0.9382	0.9335	0.9775	0.9814	0.9621	0.8663	0.9171	0.9359	0.7849	0.9472	0.9923	1.0000	0.9246
2016 NO_2_	0.8574	0.8665	0.8737	0.9282	0.8727	0.8935	0.8741	0.9716	0.8749	0.9082	0.9219	0.8862	0.9012	0.9236	0.9246	1.0000
Average	0.7099	0.7352	0.7239	0.7037	0.5884	0.6384	0.6307	0.5896	0.5113	0.5629	0.5537	0.6304	0.5722	0.6222	0.6143	0.5613

**Table 8:** Health expenditure, death rate, and phthisis efficiencies.

DMU	Region	2013Health Exp.	2014 Health Exp.	2015 Health Exp.	2016 Health Exp.	2013 Death	2014 Death	2015 Death	2016 Death	2013 Phthisis	2014 Phthisis	2015 Phthisis	2016 Phthisis
Beijing	East	1.0000	1.0000	1.0000	1.0000	1.0000	1.0000	1.0000	1.0000	1.0000	1.0000	1.0000	1.0000
Tianjin	East	1.0000	1.0000	1.0000	1.0000	1.0000	1.0000	1.0000	1.0000	1.0000	1.0000	1.0000	1.0000
Hebei	East	0.2350	0.2877	0.2114	0.1627	0.7115	0.8899	0.7539	0.7043	1.0000	1.0000	0.9273	0.8311
Liaoning	East	0.2333	0.2386	0.2236	0.1564	0.4414	0.4462	0.3992	0.3983	0.5769	0.6532	0.6411	0.4374
Shanghai	East	1.0000	1.0000	1.0000	1.0000	1.0000	1.0000	1.0000	1.0000	1.0000	1.0000	1.0000	1.0000
Jiangsu	East	0.3357	0.2496	0.2388	0.2070	0.8119	0.5502	0.5457	0.5939	1.0000	0.8498	0.8342	0.9292
Zhejiang	East	0.2213	0.2295	0.2141	0.1847	0.7707	0.7779	0.7850	0.7653	0.9585	1.0000	1.0000	0.7410
Fujian	East	0.4321	1.0000	1.0000	1.0000	0.8835	1.0000	1.0000	1.0000	1.0000	1.0000	1.0000	1.0000
Shandong	East	0.4650	1.0000	1.0000	1.0000	1.0000	1.0000	1.0000	1.0000	1.0000	1.0000	1.0000	1.0000
Guangdong	East	0.2766	0.4966	0.2626	0.2296	1.0000	1.0000	1.0000	1.0000	0.5263	0.6614	1.0000	1.0000
Hainan	East	1.0000	1.0000	1.0000	1.0000	1.0000	1.0000	1.0000	1.0000	1.0000	1.0000	1.0000	1.0000
Shanxi	Central	0.2912	0.3208	0.2775	0.1962	0.7440	0.6691	0.6848	0.6538	0.9937	1.0000	0.9755	0.8462
Jilin	Central	0.2057	0.2568	0.2445	0.1658	0.4515	0.4441	0.4491	0.3894	0.5251	0.6829	0.6677	0.4532
Heilongjiang	Central	0.1900	0.2198	0.2010	0.1286	0.4756	0.4760	0.3824	0.3361	0.4778	0.5166	0.4343	0.2344
Anhui	Central	0.2300	0.2560	0.2547	0.1610	0.8245	0.8291	0.8484	0.7679	1.0000	1.0000	1.0000	0.6839
Jiangxi	Central	0.3431	0.3599	0.3544	0.2612	0.8294	0.8256	0.8478	0.8449	1.0000	1.0000	1.0000	0.8499
Henan	Central	0.2159	0.2363	0.2104	0.1598	0.7406	0.7271	0.7281	0.6879	0.9534	1.0000	1.0000	0.6829
Hubei	Central	0.2451	0.2687	0.2411	0.2032	0.7587	0.7127	0.7822	0.7529	0.8119	0.9259	0.8762	0.8944
Hunan	Central	0.2521	0.2694	0.2718	0.1928	0.8118	0.8216	0.8248	0.7901	0.9421	0.9918	1.0000	0.8486
Inner Mongoria	West	0.3190	0.3800	0.3128	0.2110	0.6624	0.6376	0.5938	0.5626	0.9267	1.0000	0.8955	0.5623
Guangxi	West	0.3137	0.3420	0.3528	0.2584	0.9375	0.9446	0.9554	0.9562	0.9614	0.9108	1.0000	0.8745
Chongqing	West	0.2814	0.3082	0.3038	0.2759	0.6468	0.6323	0.6442	0.6879	0.8217	0.9009	0.9883	0.9236
Sichuan	West	0.1787	0.1912	0.1815	0.1275	0.6061	0.6119	0.6257	0.5954	0.7740	0.8970	0.9349	0.6449
Guizhou	West	0.3954	0.3706	0.3744	0.3095	0.7648	0.7565	0.7555	0.7680	0.6522	0.6671	0.7151	0.5261
Yunnan	West	0.2771	0.2972	0.2849	0.1922	0.7348	0.7170	0.7582	0.7371	1.0000	1.0000	1.0000	0.9171
Shaanxi	West	0.2253	0.2478	0.2413	0.1811	0.6730	0.6485	0.6544	0.6560	1.0000	1.0000	1.0000	0.7887
Gansu	West	0.4999	0.5231	0.4802	0.3814	0.8060	0.7758	0.7752	0.7856	1.0000	1.0000	1.0000	1.0000
Qinghai	West	1.0000	1.0000	1.0000	1.0000	1.0000	1.0000	1.0000	1.0000	1.0000	1.0000	1.0000	1.0000
Ningxia	West	1.0000	1.0000	1.0000	1.0000	1.0000	1.0000	1.0000	1.0000	1.0000	1.0000	1.0000	1.0000
Xinjiang	West	1.0000	1.0000	1.0000	1.0000	1.0000	1.0000	1.0000	1.0000	1.0000	1.0000	1.0000	1.0000
Average Efficiency	East	0.5635	0.6820	0.6500	0.6309	0.8745	0.8786	0.8622	0.8602	0.9147	0.9240	0.9457	0.9035
	Central	0.2467	0.2735	0.2569	0.1836	0.7045	0.6882	0.6934	0.6529	0.8380	0.8896	0.8692	0.6867
	West	0.4991	0.5146	0.5029	0.4488	0.8029	0.7931	0.7966	0.7954	0.9215	0.9433	0.9576	0.8397

**Table 9:** Health, Dealh, and Phthisis Efficiency Value Correlation Test from 2013 to 2016.

	2013 Health	2014 Health	2015 Health	2016 Health	2013 Death	2014 Death	2015 Death	2016 Death	2013 Phthisis	2014 Phthisis	2015 Phthisis	2016 Phthisis
2013 Health	1.0000	0.9051	0.9104	0.9089	0.7292	0.6889	0.6750	0.6976	0.4123	0.3634	0.3401	0.5767
2014 Health	0.9051	1.0000	0.99160	0.9912	0.7751	0.7878	0.7678	0.7851	0.4002	0.3721	0.3886	0.6369
2015 Health	0.9104	0.9916	1.0000	0.9978	0.7489	0.7577	0.7434	0.7622	0.4365	0.3984	0.3730	0.6116
2016 Health	0.9090	0.9912	0.9980	1.0000	0.7497	0.7570	0.7426	0.7629	0.4251	0.3888	0.3716	0.6187
2013 Death	0.7292	0.7751	0.7489	0.7497	1.0000	0.9364	0.9523	0.9636	0.5900	0.5308	0.6992	0.8362
2014 Death	0.6890	0.7878	0.7577	0.7570	0.9364	1.0000	0.9822	0.9703	0.5425	0.5336	0.6878	0.7672
2015 Death	0.6750	0.7678	0.7439	0.7426	0.9523	0.9822	1.0000	0.9911	0.5613	0.5678	0.7436	0.8085
2016 Death	0.6976	0.7851	0.7622	0.7629	0.9636	0.9703	0.9911	1.0000	0.5620	0.5491	0.7412	0.8441
2013 Phthisis	0.4123	0.4002	0.4365	0.4251	0.5900	0.5425	0.5613	0.5620	1.0000	0.9455	0.7839	0.6826
2014 Phthisis	0.3634	0.3721	0.3984	0.3887	0.5308	0.5336	0.5678	0.5491	0.9455	1.0000	0.8568	0.6671
2015 Phthisis	0.3401	0.3886	0.3730	0.3716	0.6992	0.6878	0.7436	0.7412	0.7839	0.8568	1.0000	0.8242
2016 Phthisis	0.5767	0.6369	0.6116	0.6187	0.8362	0.7672	0.8085	0.8441	0.6826	0.6671	0.8242	1.0000
Average	0.4554	0.5117	0.4913	0.4448	0.8029	0.7967	0.7931	0.7811	0.8967	0.9219	0.9297	0.8223

**Table 10:** Efficiency characteristics of health treatment stage variables.

DMU	Health Expenditures	Death Rate	Phthisis	Characteristic
Beijing	1.0000	1.0000	1.0000	No room for improvement, best efficiency
Tianjin	1.0000	1.0000	1.0000	No room for improvement, best efficiency
Hebei	0.2242	0.7649	0.9396	There is a lot of room for improvement in health expenditures, there is some room for improvement in the death rate, and less room for improvement in phthisis
Shanxi	0.2714	0.6879	0.9539	There is a lot of room for improvement in health expenditures, there is some room for improvement in the death rate, and less room for improvement in phthisis
Inner Mongoria	0.3057	0.6141	0.8461	There is a lot of room for improvement in health expenditures, and there is some room for improvement in both the death rate and phthisis
Liaoning	0.2130	0.4213	0.5771	There is a lot of room for improvement in health expenditures and the death rate, and there is some room for improvement in phthisis
Jilin	0.2182	0.4335	0.5822	There is a lot of room for improvement in health expenditures and the death rate, and there is some room for improvement in phthisis
Heilongjiang	0.1849	0.4175	0.4158	There is a lot of room for improvement in each variable
Shanghai	1.0000	1.0000	1.0000	No room for improvement; at best efficiency
Jiangsu	0.2578	0.6254	0.9033	There is a lot of room for improvement in health expenditures, there is some room for improvement in the death rate, and less room for improvement in phthisis
Zhejiang	0.2124	0.7747	0.9249	There is a lot of room for improvement in health expenditures, there is some room for improvement in the death rate, and less room for improvement in phthisis
Anhui	0.2254	0.8175	0.9210	There is a lot of room for improvement in health expenditures, there is some room for improvement in the death rate, and less room for improvement in phthisis
Fujian	0.8580	0.9709	1.0000	There is some room for improvement in health expenditures, there is little room for improvement in the death rate, and no room for improvement in phthisis
Jiangxi	0.3296	0.8369	0.9625	There is a lot of room for improvement in health expenditures, there is some room for improvement in the death rate, and less room for improvement in phthisis
Shandong	0.8662	1.0000	1.0000	There is some room for improvement in health expenditures, and there is no room for improvement in the death rate and phthisis
Henan	0.2056	0.7209	0.9091	There is a lot of room for improvement in health expenditures, there is some room for improvement in the death rate, and less room for improvement in phthisis
Hubei	0.2395	0.7516	0.8771	There is a lot of room for improvement in health expenditures, and there is some room for improvement in both the death rate and phthisis
Hunan	0.2465	0.8121	0.9456	There is a lot of room for improvement in health expenditures, there is some room for improvement in the death rate, and less room for improvement in phthisis
Guangdong	0.3163	1.0000	0.7969	There is a lot of room for improvement in health expenditures, there is no room for improvement in the death rate, and phthisis has some room for improvement.
Guangxi	0.3167	0.9484	0.9367	There is much room for improvement in health expenditures, and there is less room for improvement in the death rate and phthisis
Hainan	1.0000	1.0000	1.0000	No room for improvement; at best efficiency
Chongqing	0.2923	0.6528	0.9086	There is a lot of room for improvement in health expenditures, there is some room for improvement in the death rate, and less room for improvement in phthisis
Sichuan	0.1697	0.6098	0.8127	There is a lot of room for improvement in health expenditures, there is some room for improvement in the death rate, and less room for improvement in phthisis
Guizhou	0.3625	0.7612	0.6401	There is a lot of room for improvement in health expenditures, and there is some room for improvement in both the death rate and phthisis
Yunnan	0.2629	0.7368	0.9793	There is a lot of room for improvement in health expenditures, there is some room for improvement in the death rate, and less room for improvement in phthisis
Shaanxi	0.2239	0.6580	0.9472	There is a lot of room for improvement in health expenditures, there is some room for improvement in the death rate, and less room for improvement in phthisis
Gansu	0.4711	0.7856	1.0000	There is a lot of room for improvement in health expenditures, there is some room for improvement in the death rate, and no room for improvement in phthisis
Qinghai	1.0000	1.0000	1.0000	No room for improvement; at best efficiency
Ningxia	1.0000	1.0000	1.0000	No room for improvement; at best efficiency
Xinjiang	1.0000	1.0000	1.0000	No room for improvement; at best efficiency
